# Glycosylation-driven necroptosis in retinal degeneration: dual rescue by AAV8 gene therapy and RIPK1 inhibition

**DOI:** 10.1038/s41420-026-03098-8

**Published:** 2026-04-09

**Authors:** Jia-Ying Chien, Peng Yeong Woon, Hsien-Yang Tsai, Mei-Ling Peng, Shi-Huang Lee, Siu-Fung Chau, Yu-Chen Chen, Wai-Man Cheang, Ching-Yen Tsai, Shun-Ping Huang

**Affiliations:** 1 Department of Biochemical Science and Technology, National Chiayi University, Chiayi City, Taiwan; 2 Molecular Biology and Human Genetics, Tzu Chi University, Hualien, Taiwan; 3 Department of Ophthalmology, Taichung Tzu Chi Hospital, Taichung, Taiwan; 4 Transgenic Core Facility, Institute of Molecular Biology, Academia Sinica, Taipei, Taiwan

**Keywords:** Hereditary eye disease, Gene expression, Cell death in the nervous system

## Abstract

Glycosylation defects are increasingly implicated across neurodegenerative diseases, yet the mechanism by which perturbed O-mannosylation drives neuronal death—and how to reverse it—remains unclear. Here we show that a disease-associated *POMGnT1* L120R mutation produces widespread retinal neurodegeneration by coupling metabolic collapse to necroptosis. In mice harboring the human *POMGnT1* L120R allele and in *POMGnT1*-knockout human RPE cells, hypoglycosylation of key substrates (α-dystroglycan and ENO1) coincides with strengthened SAG–ENO1 interaction, reduced glycolytic capacity, ATP shortfall, Golgi fragmentation, tight-junction failure, and robust activation of the RIPK1/RIPK3/MLKL cascade; notably, degeneration proceeds with minimal apoptotic signatures. Two orthogonal interventions—AAV8-mediated *POMGnT1* gene augmentation and pharmacologic RIPK1 inhibition (RIPA-56)—each suppress necroptotic signaling, restore barrier integrity, and rescue visual function in vivo. These data define a glycosylation-metabolism-necroptosis axis that generalizes beyond a single gene or tissue and motivate a mutation-independent therapeutic blueprint: repair the upstream glycosylation deficit and/or block the downstream necroptotic execution pathway. Our findings position O-mannosylation homeostasis as a tractable control point for neuroprotection and nominate combined gene-augmentation and kinase-inhibition strategies for glycosylation-linked neurodegeneration.

## Background

Protein O-linked-mannose beta-1,2-N-acetylglucosaminyltransferase 1 (POMGnT1) is an enzyme that participates in the synthesis of O-mannosyl glycans encoded by the *POMGnT1* gene in humans [1]. O-mannosyl glycosylation is a dynamic posttranslational modification involved in glycosylation that mediates synaptic neurotransmitter release and transporters in the central nervous system (CNS), thereby affecting CNS development and function [2–4]. The vital function of POMGnT1 is to facilitate the addition of N-acetylglucosamine (GlcNAc) to O-linked mannose in glycoproteins. This process is essential for maintaining the structural integrity and function of proteins in the nervous system and retina.

POMGnT1 is expressed in the inner segments, outer plexiform layer (OPL), and inner limiting membrane (ILM) of mammalian photoreceptors, where it colocalizes with GM130, a Golgi complex marker in the retina. Mutations in POMGnT1 in humans cause nonsyndromic retinitis pigmentosa (NSRP), congenital muscular dystrophy (CMD), and muscle-eye-brain (MEB) disease [5]. Patients with mutant *POMGnT1* have a variety of retinal dystrophies, including glaucoma, nystagmus, electroretinography (ERG) abnormalities, retinal detachment, and dysplasia. Moreover, fundus photography revealed detached retinas covered with blood vessels in POMGnT1-deficient mice. Histological examination confirmed a thinner retina and higher GFAP expression in the eyes of POMGnT1-deficient mice than in those of control mice [6–9]. Furthermore, a missense mutation in *POMGNT1* (c.359 A > C, p.Leu120Arg, and L120R) is involved in autosomal recessive retinitis pigmentosa (RP). This mutant POMGNT1 protein causes an approximately 80% decrease in enzyme activity in the retina [7]. These findings suggest that POMGNT1 is indispensable in the retina and the nervous system.

Although a genetic association between *POMGnT1* mutations and NSRP has been established, the precise mechanisms through which these mutations cause retinal degeneration remain unclear. POMGnT1 is expressed in photoreceptor cells, suggesting a critical functional role. However, its exact pathological impact on retinal degeneration is not fully understood. To address these gaps, we investigated the molecular and cellular roles of POMGnT1 in retinal function using a mutant Pomgnt1 mouse model and POMGnT1-knockout human retinal pigment epithelial (RPE) cell lines. We further evaluated the therapeutic potential of adeno-associated virus serotype 8 vector (AAV8)-mediated gene delivery by introducing the human *POMGnT1* gene into both models. Our results highlight a novel therapeutic strategy for RP caused by POMGnT1 loss of function, offering insights into retinal biology. Moreover, we found that the L120R mutant protein stabilizes the SAG–ENO1 complex, leading to inflammation, reduced glycolysis, ATP depletion, and necroptosis through RIP3 and MLKL activation, along with autophagy inhibition. Importantly, treatment with RIPA-56, a RIP1 kinase (RIPK1) inhibitor, rescued retinal function in *Pomgnt1*^L120R/L120R^ mice. These findings expand our understanding of the role of POMGnT1 in retinal degeneration and identify potential therapeutic targets for treating retinal dystrophies.

## Results

### Age-dependent rod photoreceptor dysfunction and scotopic ERG abnormalities in Pomgnt1^L120R/L120R^ mutant mice

**Fig. 1:**
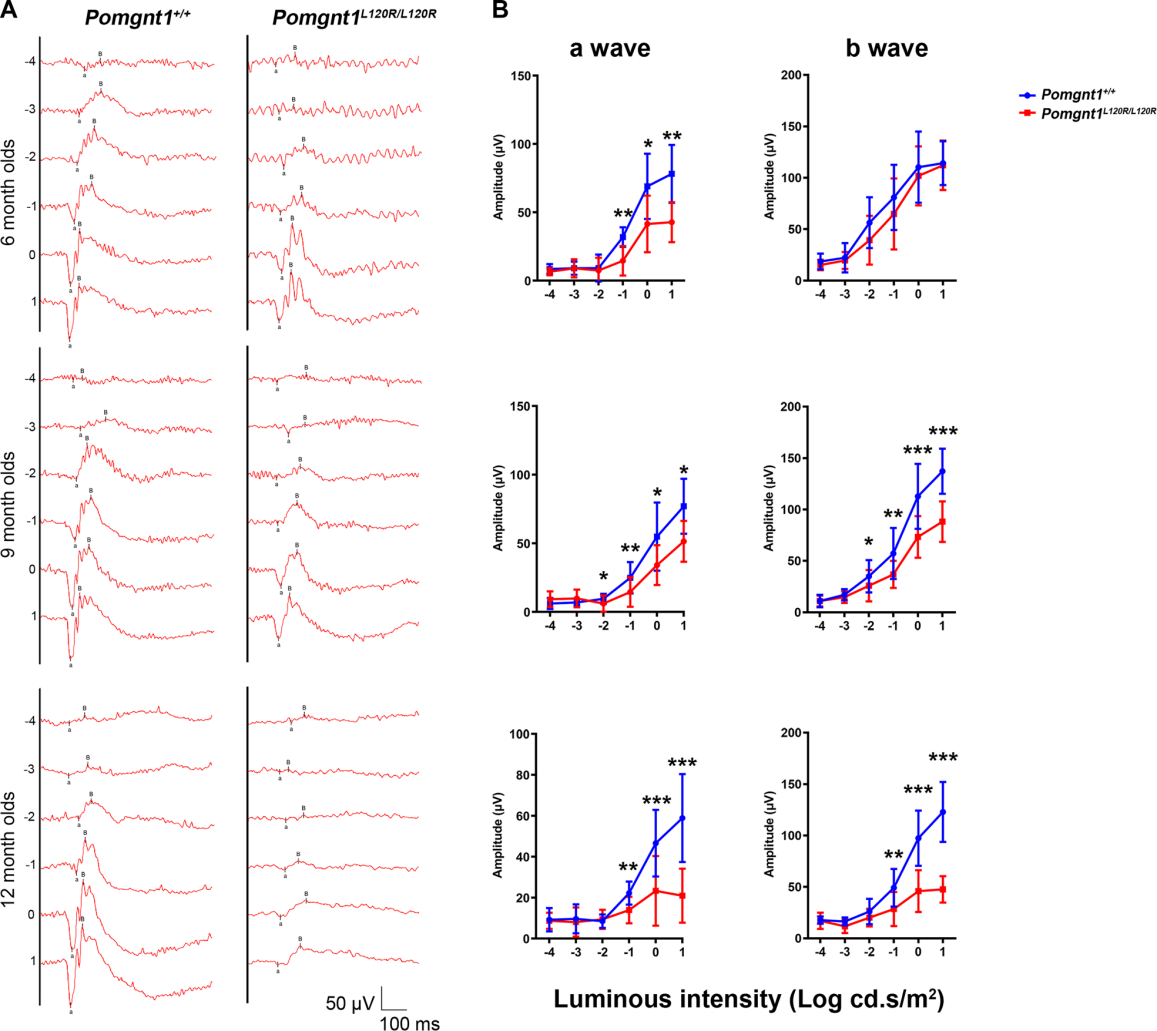
Scotopic ERG analysis of *Pomgnt1*^*L120R/L120R*^ and *Pomgnt1*^*+/+*^ mice. **A** Representative photopic electroretinogram (ERG) waveforms were recorded for *Pomgnt1*^*L120R/L120R*^ and *Pomgnt1*^*+/+*^ mice at 6, 9, and 12 months of age. **B** Quantitative analysis of a-wave and b-wave amplitudes revealed a significant reduction in the *Pomgnt1*^*L120R/L120R*^ group compared with the *Pomgnt1*^*+/+*^ control group across all time points. Statistical comparisons revealed progressive decreases in the mutant group, with significance levels denoted as *, *P* < 0.05; **, *P* < 0.01; ***, *P* < 0.001. The data are presented as the means ± standard deviations (SDs). (*n* = 12 per group).

Patients harboring *POMGnT1* homozygous L120R mutations exhibit reduced or absent scotopic ERG b-waves but notably lack associated neurological manifestations such as intellectual disability, muscle weakness, and atrophy [5]. Consistent with these findings, the hindlimb extension test revealed no evidence of muscle weakness or atrophy in *Pomgnt1*^*L120R/L120R*^ mice (data not shown). To assess retinal function in vivo, ERG was performed under dark-adapted scotopic conditions using a range of light stimulus intensities (0.0001–10 cds/m^2^). Scotopic ERG measures include the a-wave, reflecting rod photoreceptor activity, and the b-wave, reflecting rod-to-rod bipolar cell synaptic transmission. Scotopic ERG recordings revealed significant rod dysfunction, as evidenced by the reduced a- and b-wave amplitudes in *Pomgnt1*^*L120R/L120R*^ mice at both 6 and 9 months of age (Fig. 1). The progressive decline in wave amplitudes suggests an age-dependent impairment of rod photoreceptor function and postsynaptic signal transmission in rod bipolar cells within the mutant mouse retina.

### Age-dependent retinal degeneration and ultrastructural alterations in Pomgnt1^L120R/L120R^ mice

**Fig. 2:**
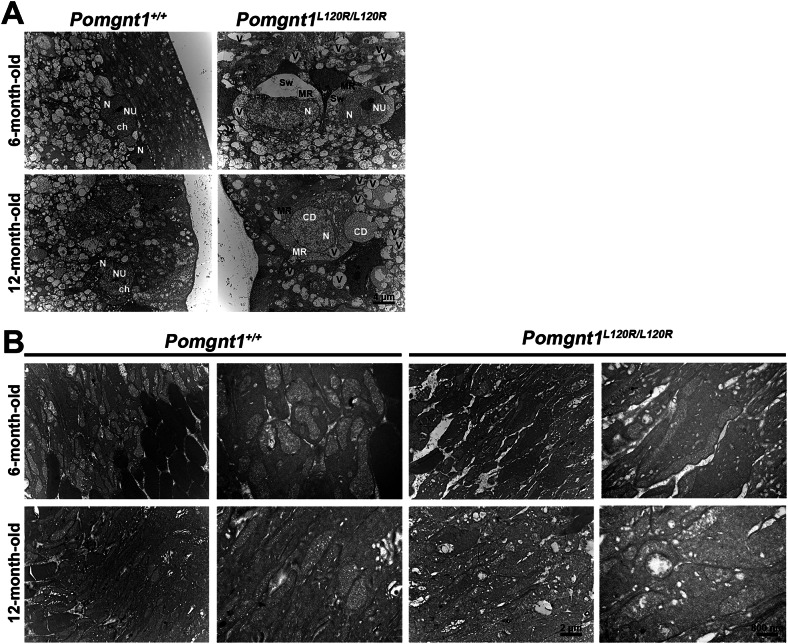
Microstructural alterations in retinal layers and mitochondrial morphology in *Pomgnt1*^*L120R/L120R*^ mice. **A** TEM analysis revealed significant pathological changes in the retinal GCL in *Pomgnt1*^*L120R/L120R*^ mice compared with *Pomgnt1*^*+/+*^ controls. Retinal ganglion cells in the mutant mice exhibited pronounced swelling and membrane rupture at 6 and 12 months of age. Scale bars: 4 μm. N (nucleus), NU (nucleoli), V (cytoplasmic vacuolization), Ch (chromatin), CD (chromatin dissolution), MR (membrane rupture), and Sw (swelling). **B** TEM analysis of mitochondrial morphology revealed progressive vacuolization and structural abnormalities in *Pomgnt1*^*L120R/L120R*^ mice, with more severe defects observed at 12 months of age. Scale bars: 2 μm and 800 nm (*n* = 6 per group).

Transmission electron microscopy (TEM) was used to examine the retinas of *Pomgnt1*^*L120R/L120R*^ mice at 6 and 12 months of age. At 6 months of age, TEM analysis of the nuclei ultrastructure in the ganglion cell layer (GCL) revealed a reduction in nuclear matrix density in the mutant mice compared with the wild-type controls (*Pomgnt1*^*+/+*^). By 12 months of age, *Pomgnt1*^*L120R/L120R*^ mouse retinas presented pronounced ultrastructural abnormalities, including chromatin dissolution, vacuolization of cytoplasmic organelles, membrane rupture, and swollen cells in the GCL (Fig. 2A). TEM analysis further revealed vacuolated mitochondria and disrupted crista architecture within the photoreceptor inner segments (ISs) of *Pomgnt1*^*L120R/L120R*^ retinas at 12 months of age (Fig. 2B). Histological analysis corroborated these findings, revealing a significant reduction in retinal thickness in mutant mice at 12 months, with a particularly notable thinning of the outer nuclear layer (ONL) compared with the retinal thickness in wild-type mice (Supplementary Fig. 2). Morphological assessment revealed irregularly shaped and vacuolated mitochondria and retinal ganglion cells in *Pomgnt1*^*L120R/L120R*^ retinas at 12 months of age. These structural abnormalities provide evidence of severe retinal degeneration and mitochondrial dysfunction in the *Pomgnt1*^*L120R/L120R*^ model, potentially contributing to disease progression.

### Increased numbers of astrocytes and Müller cells in the retinas of Pomgnt1^L120R/L120R^ mice

**Fig. 3:**
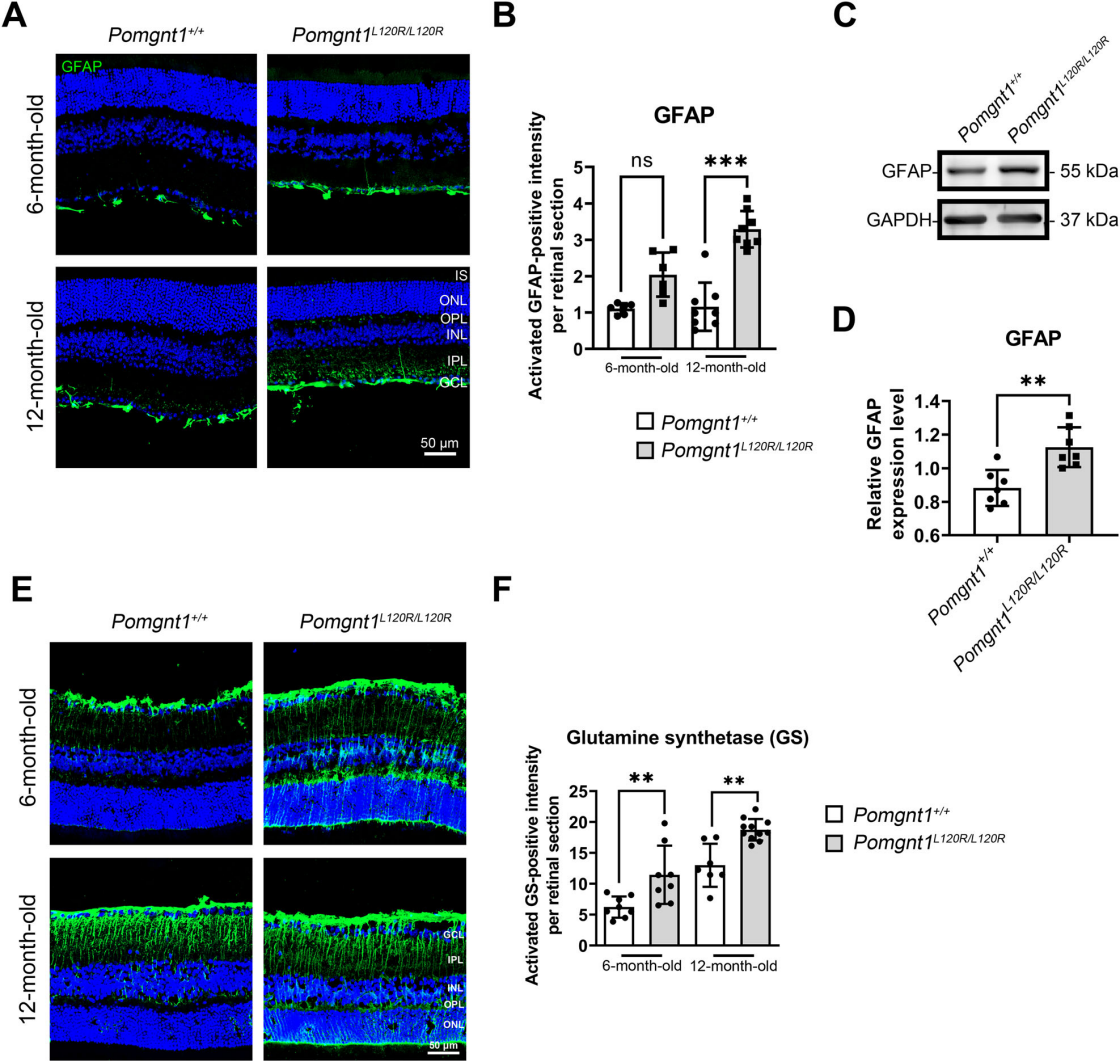
Reactive gliosis and upregulation of GFAP and GS in the retinas of *Pomgnt1*^*L120R/L120R*^ mice. **A** Immunofluorescence staining of GFAP (green) in retinal sections from *Pomgnt1*^*+/+*^ and *Pomgnt1*^*L120R/L120R*^ mice at 6 and 12 months of age. GFAP expression is markedly elevated in the GCL and extends into the inner nuclear layer (INL) of Pomgnt1^L120R/L120R^ mouse retinas. Nuclei were counterstained with DAPI (blue). Scale bar: 50 µm. **B** Quantification of GFAP-positive staining in retinal sections revealed a significant increase in GFAP staining intensity in 12-month-old *Pomgnt1*^*L120R/L120R*^ mice compared with controls. **C** Immunoblot analysis of GFAP expression in retinal lysates from *Pomgnt1*^*+/+*^ and *Pomgnt1*^*L120R/L120R*^ mice. GAPDH was used as a loading control. **D** Relative quantification of GFAP protein levels normalized to the level of GAPDH, demonstrating significant GFAP upregulation in *Pomgnt1*^*L120R/L120R*^ mouse retinas. (*, *p* < 0.05; **, *p* < 0.01; *n* = 7) **E** Immunofluorescence staining of GS (green) in retinal sections from *Pomgnt1*^*+/+*^ and *Pomgnt1*^*L120R/L120R*^ mice. GS expression, which is confined to Müller glial cells, was significantly elevated in the *Pomgnt1*^*L120R/L120R*^ retina at 6 months of age and further increased by 12 months of age, with processes extending into the ONL. Nuclei were counterstained with DAPI (blue). Scale bar: 50 µm. **F** Quantification of GS-positive staining per retinal section, demonstrating that GS was significantly upregulated in *Pomgnt1*^*L120R/L120R*^ mice at both 6 and 12 months of age. (Scale bar = 50 µm; *, *p* < 0.05; **, *p* < 0.01; *n* = 6).

To investigate retinal gliosis responses associated with photoreceptor degeneration, glial fibrillary acidic protein (GFAP) was examined as a marker of retinal gliosis in astrocytes during retinal degeneration. Under normal conditions, GFAP immunoreactivity is confined to the retinal GCL [10]. In retinal sections from 12-month-old *Pomgnt1*^*L120R/L120R*^ mice, marked upregulation of GFAP expression was observed in the GCL, with GFAP-positive processes extending into the inner nuclear layer (INL) (Fig. 3A–D). Glutamate accumulation in neurons, which is often observed during CNS injury, induces excitotoxicity and neuronal degeneration. Müller glial cells, the principal glial cells in the retina, play a protective role by mitigating glutamate toxicity. They achieve this by upregulating glutamine synthetase (GS), which converts glutamate to glutamine and activates endogenous neuroprotective mechanisms to combat neuronal degeneration [11]. While GS upregulation is neuroprotective, excessive GS expression can result in neuronal hyperexcitability and cellular edema [12, 13]. In the *Pomgnt1*^*L120R/L120R*^ mouse retina, GS immunoreactivity was significantly elevated in the GCL and extended into the INL by six months of age. By 12 months of age, GS expression further increased, with Müller cell processes reaching the outer nuclear layer (ONL) (Fig. 3E, F). These findings suggest that neuronal degeneration accompanied by reactive gliosis is a prominent feature of the retinas of *Pomgnt1*^*L120R/L120R*^ mice.

### POMGnT1 interacts with enolase-1 and S-arrestin in the mouse retina

**Fig. 4:**
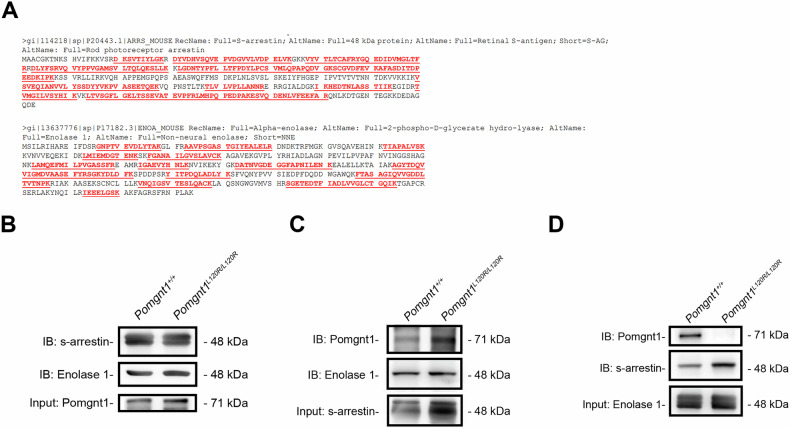
Aberrant interactions among POMGnT1, enolase-1, and S-arrestin in the *Pomgnt1*^*L120R/L120R*^ mouse retina. **A** Sequence alignment of the SAG and ENO1 proteins identified via mass spectrometry in retinal lysates immunoprecipitated with an anti-POMGnT1 antibody. The identified peptides are highlighted in red. **B** Coimmunoprecipitation of POMGnT1-associated proteins from the retinal lysates of *Pomgnt1*^*+/+*^ and *Pomgnt1*^*L120R/L120R*^ mice. Immunoblots showing successful pull-down of s-arrestin and enolase-1 in both genotypes using anti-POMGnT1 antibodies. **C** Immunoprecipitation with anti-s-arrestin antibodies revealed interactions between enolase-1 and POMGnT1 in both *Pomgnt1*^*+/+*^ and *Pomgnt1*^*L120R/L120R*^ retinal lysates. **D** Immunoprecipitation with anti-enolase-1 antibodies revealed differential interactions with POMGnT1. POMGnT1 was detected in *Pomgnt1*^*+/+*^ mouse retinal lysates but was absent in *Pomgnt1*^*L120R/L120R*^ mouse retinal lysates. S-Arrestin was detected in retinal lysates from both mouse groups.

To identify the proteins that interact with POMGnT1 in the retina, we performed pull-down assays using retinal lysates from *Pomgnt1*^*+/+*^ and *Pomgnt1*^*L120R/L120R*^ mice. Immunoprecipitation was conducted with a mouse monoclonal antibody against POMGnT1 (Cat# WH0055624M7), followed by SDS‒PAGE and mass spectrometry. The identified protein bands were confirmed as enolase-1 (ENO1) and s-arrestin (SAG) (Fig. 4A). Coimmunoprecipitation assays were performed to further validate the interactions in retinal homogenates from *Pomgnt1*^*+/+*^ and *Pomgnt1*^*L120R/L120R*^ mice. Immunoprecipitation reactions targeting POMGnT1-associated proteins confirmed the interactions among POMGnT1, ENO1, and SAG in both genotypes (Fig. 4B). Additional experiments targeting s-arrestin revealed that antibodies against POMGnT1 and ENO1 successfully detected their respective targets in retinal lysates from both genotypes (Fig. 4C). However, when ENO1 was immunoprecipitated from retinal lysates, subsequent immunoblotting with anti-SAG and anti-POMGnT1 antibodies produced different results. While SAG was detected in lysates from both *Pomgnt1*^*+/+*^ and *Pomgnt1*^*L120R/L120R*^ mice (Fig. 4c), POMGnT1 was detected only in *Pomgnt1*^*+/+*^ mouse retinal lysates but not in *Pomgnt1*^*L120R/L120R*^ mouse retinal lysates (Fig. 4D).

To determine whether the L120R mutation directly alters binding affinity, we next performed GST pull-down assays using purified recombinant human proteins. GST-ENO1 precipitated FLAG-tagged S-arrestin and His-tagged wild-type POMGNT1, demonstrating direct biochemical interactions. In contrast, His-tagged POMGNT1-L120R failed to bind GST-ENO1, indicating that the mutation disrupts the POMGNT1–ENO1 interface (Supplementary Fig. 7).

Together, the in vitro and in vivo evidence demonstrates that the L120R mutation selectively impairs POMGnT1 binding to the ENO1–SAG complex, potentially destabilizing this molecular assembly in the retina. Such disruption may contribute to enhanced inflammatory responses and increased susceptibility to retinal cell death in POMGnT1-deficient conditions.

### Altered retinal glycoprotein profiles in Pomgnt1^L120R/L120R^ mice

**Fig. 5:**
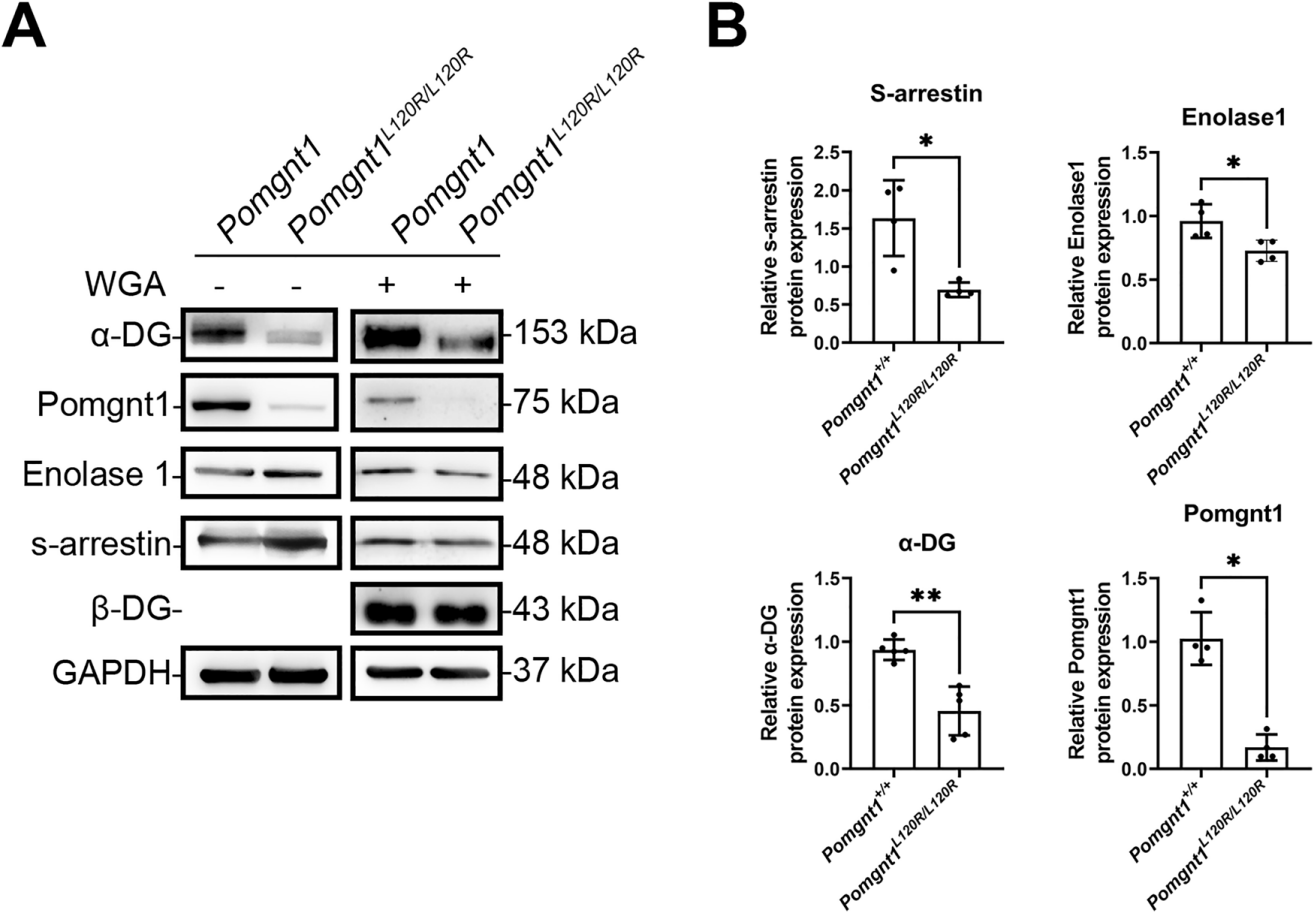
Altered glycoprotein profiles in *Pomgnt1*^*L120R/L120R*^ mutant mouse retinas. **A** Western blot analysis of retinal lysates from WT and *Pomgnt1*^L120R/L120R^ mutant mice, showing the expression and glycosylation status of key proteins. Samples were analyzed with and without wheat germ agglutinin (WGA) enrichment to assess glycoprotein levels. Decreased levels of α-dystroglycan (α-DG), POMGnT1, and glycosylated enolase-1 (ENO1) are observed in *Pomgnt1*L120R/L120R retinas. S-arrestin (SAG) protein levels are increased in the mutant retinas. GAPDH serves as a loading control. Molecular weights are indicated on the right. **B** Quantitative analysis of relative protein expression levels from Western blot data shown in (**A**), normalized to GAPDH. Bar graphs illustrate the significant reductions in the relative glycosylated protein expression levels of enolase-1, α-DG, and POMGnT1 in *Pomgnt1*^L120R/L120R^ mice compared with WT controls. Data are presented as mean ± SD. Statistical significance is indicated by asterisks (*, *p* < 0.05; **, *p* < 0.01; *n* = 4).

To investigate the impact of the *Pomgnt1* L120R mutation on retinal glycosylation, glycoproteins were isolated from retinas of WT and *Pomgnt1*^*L120R/L120R*^ mice using lectin affinity purification. Western blot analysis of whole retinal lysates, with and without wheat germ agglutinin (WGA) enrichment, revealed significant changes in the glycosylation status and expression levels of key retinal proteins (Fig. 5A) Specifically, the glycosylated α-dystroglycan (α-DG) was markedly decreased in the mutant retinas, as evidenced by its reduced presence in both total lysates and WGA-enriched fractions. Furthermore, the protein expression of POMGnT1 itself was significantly reduced in the *Pomgnt1*^*L120R/L120R*^ mice. Notably, ENO1 showed a decrease in its glycosylated form in the mutant retina, whereas total ENO1 protein and SAG protein levels were increased in the mutant retinas. Quantitative analysis of WGA-enriched fractions (Fig. 5B) confirmed the significant reduction in the relative protein expression levels of S-arrestin, enolase-1, α-DG, and POMGnT1 in *Pomgnt1*^*L120R/L120R*^ mice compared with WT controls. These findings collectively indicate a broad impact of the *POMGnT1* L120R mutation on retinal glycoprotein biosynthesis and protein expression, contributing to the observed pathological phenotype.

### Necroptosis is the predominant pathway responsible for retinal degeneration in Pomgnt1^L120R/L120R^ mice

**Fig. 6:**
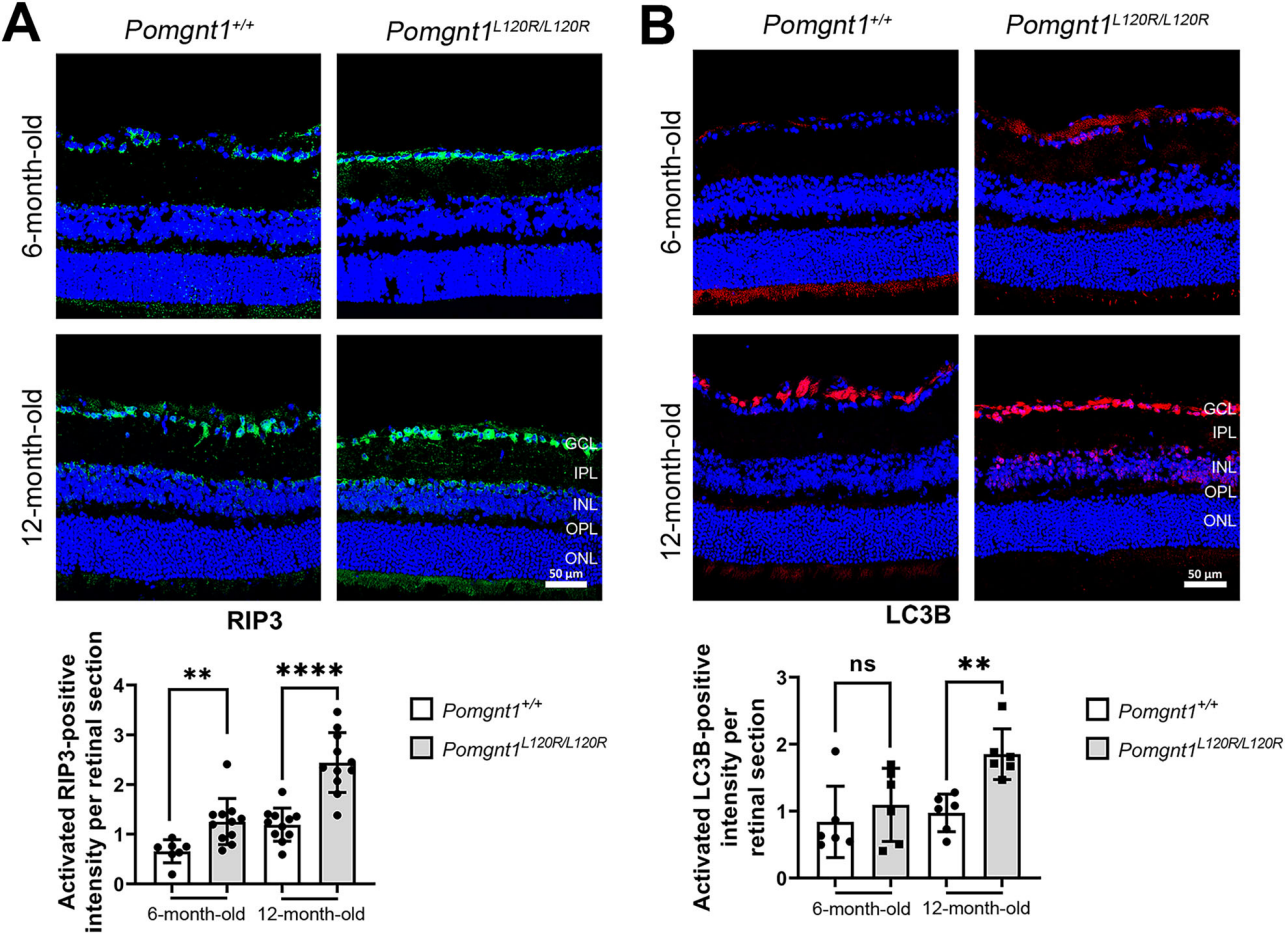
Upregulation of necroptotic and autophagic markers in *Pomgnt1*^*L120R/L120R*^ retinas. **A** Immunofluorescence analysis of the necroptotic marker RIP3 (green) in retinal cryosections from *Pomgnt1*^*+/+*^ (wild-type) and *Pomgnt1*^*L120R/L120R*^ mice at 6 and 12 months of age. Increased RIP3-positive signals were observed in the GCLs and ONLs of Pomgnt1L120R/L120R mice compared with wild-type controls, with significant upregulation at 12 months of age. The quantification of the RIP3-positive signal intensity per retinal section is shown in the lower panel. (Scale bar = 50 µm, *, *p* < 0.05; **, *p* < 0.0; ***, *p* < 0.001; *n* = 6) **B** Immunofluorescence analysis of the autophagy marker LC3B (red) in retinal cryosections of *Pomgnt1*^*+/+*^ and *Pomgnt1*^*L120R/L120R*^ mice at 6 and 12 months of age. An elevated LC3B-positive signal was observed in *Pomgnt1*^*L120R/L120R*^ mice at 12 months, particularly in the GCL and ONL. The quantification of the LC3B-positive signal intensity per retinal section is shown in the lower panel. Nuclei were counterstained with DAPI (blue). (Scale bar = 50 µm, *, *p* < 0.05; **, *p* < 0.01; ***, *p* < 0.001; n = 6).

Recent studies have identified various forms of regulated apoptosis, necrosis, and autophagy as mechanisms contributing to neurodegeneration in RP [14–18]. To elucidate the neurodegenerative pathways involved in retinal degeneration in *Pomgnt1*^*L120R/L120R*^ mice, we assessed markers of apoptosis, necroptosis, and autophagy in retinal tissue. TUNEL assays and immunodetection of the necroptotic marker RIP3 and the autophagy marker LC3B were performed. Analysis of retinal cryosections revealed a marked increase in RIP3 (Fig. 6A) and LC3B (Fig. 6B) protein expression in *Pomgnt1*^*L120R/L120R*^ mice. However, no TUNEL-positive cells were observed across retinal sections (Supplementary Fig. 3). These findings suggest that retinal degeneration in *Pomgnt1*^*L120R/L120R*^ mice occurs predominantly through necroptosis rather than through apoptosis.

### Upregulation of necroptosis and autophagy pathway members in Pomgnt1^L120R/L120R^ retinas

**Fig. 7:**
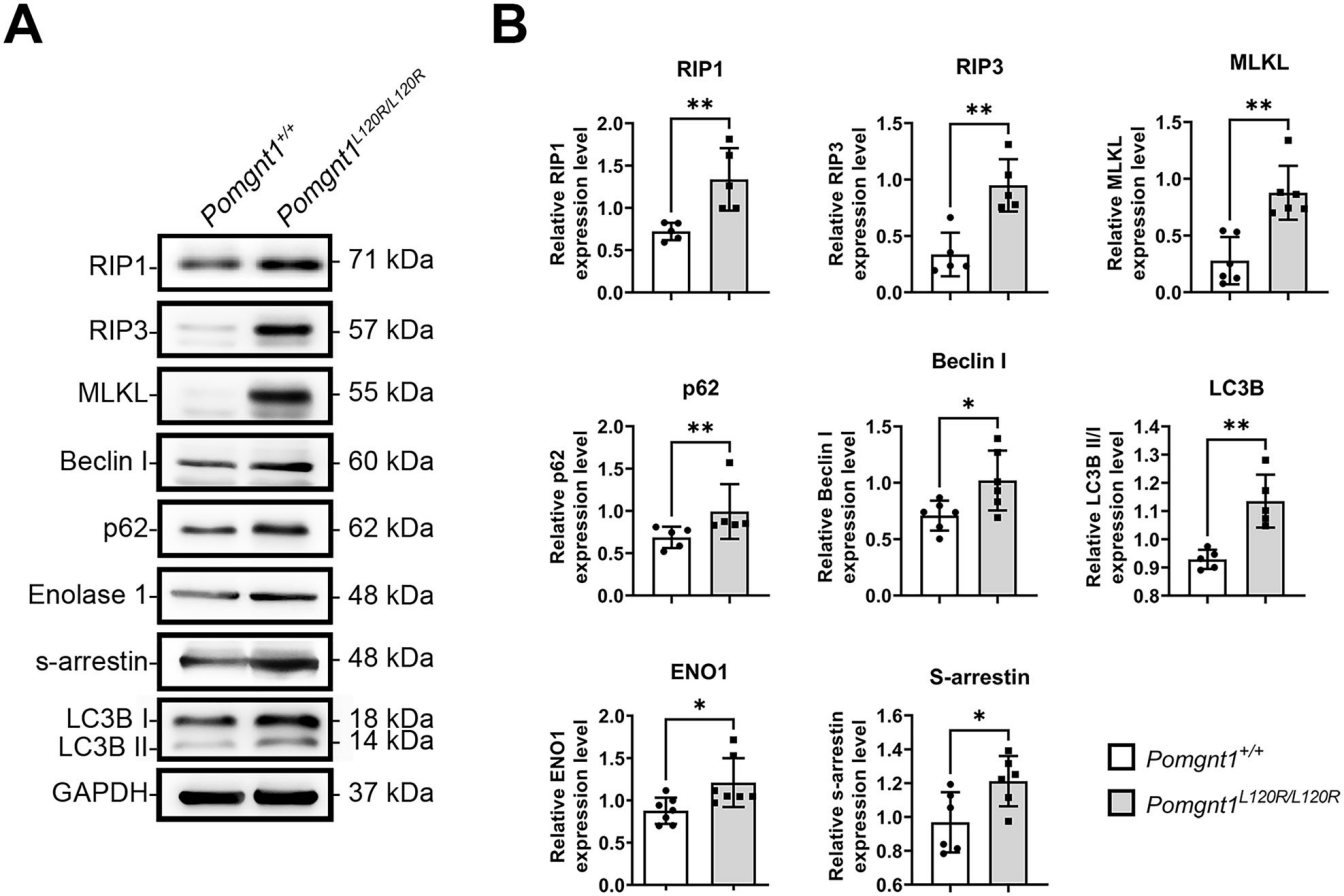
Increased expression of necroptotic and autophagic signaling markers in *Pomgnt1*^*L120R/L120R*^ mouse retinas. **A** Western blot analysis of necroptosis- and autophagy-associated protein levels in retinas from *Pomgnt1*^*+/+*^ and *Pomgnt1*^*L120R/L120R*^ mice. The proteins analyzed included RIP1, RIP3, MLKL, Beclin1, p62, enolase-1 (ENO1), S-arrestin (SAG), and LC3B. GAPDH was used as a loading control. Increased levels of RIP1, RIP3, MLKL, Beclin1, p62, ENO1, SAG, and LC3B-II were observed in *Pomgnt1*^*L120R/L120R*^ retinas, indicating increased necroptotic and autophagic signaling. **B** Quantitative analysis of Western blot data presented as relative protein expression levels normalized to those of GAPDH and expressed relative to those of *Pomgnt1*^*+/+*^ controls. Elevated levels of necroptosis-associated proteins (RIP1, RIP3, and MLKL), autophagy-related proteins (Beclin1, p62, and LC3B-II), and SAG–ENO1 complex components (ENO1 and SAG) were observed in *Pomgnt1*^*L120R/L120R*^ mouse retinas compared to control mouse retinas. (*, *p* < 0.05; **, *p* < 0.01; *n* = 12).

We investigated the molecular mechanisms underlying retinal degeneration in *Pomgnt1*^*L120R/L120R*^ mice, focusing on the expression of necroptosis- and autophagy-related proteins and their potential links to photoreceptor metabolism. Western blot analysis (Fig. 7A, B) revealed significant increases in RIP1, RIP3, and MLKL protein levels in the retinas of *Pomgnt1*^*L120R/L120R*^ mice. Notably, the expression levels of SAG and ENO1 were also elevated, as were the levels of the autophagy-associated proteins Beclin1, p62, and LC3B. The upregulation of p62, a substrate and biomarker of autophagic flux [19, 20], combined with elevated Beclin1 and LC3B levels, suggests the inhibition of autophagic flux in *Pomgnt1*^*L120R/L120R*^ mouse retinas.

### RIPA-56 attenuates necroptosis-induced cell death and restores paracellular permeability in human retinal pigment epithelial (RPE) cells by targeting the RIP1 pathway

**Fig. 8:**
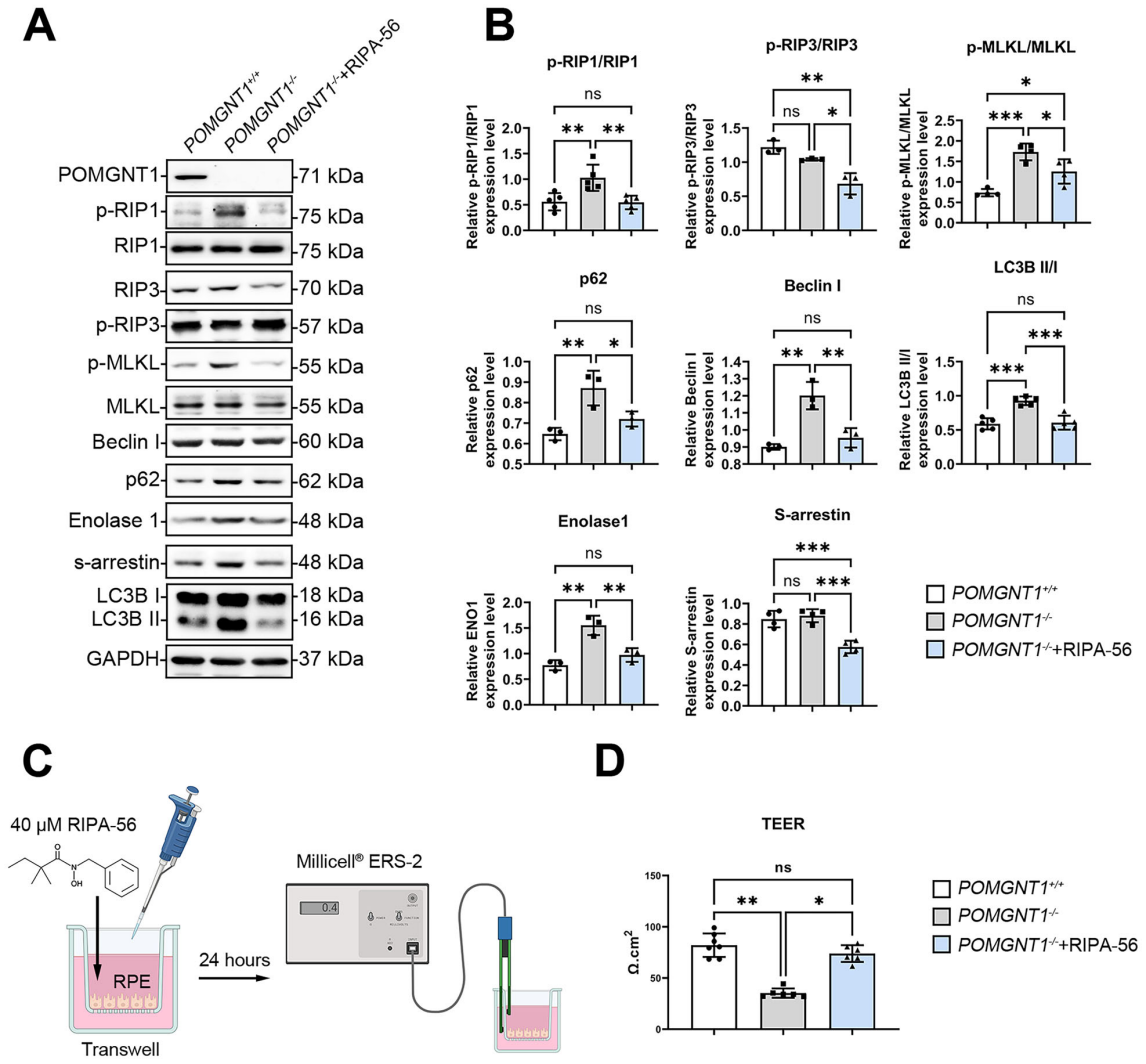
RIPA-56 inhibits the necroptosis pathway and restores barrier integrity in POMGnT1-deficient RPE cells. **A** Western blot analysis of necroptosis-associated proteins and autophagy markers in wild-type (*POMGnT1*^*+/+*^), POMGnT1 knockout (*POMGnT1*^*-/-*^), and RIPA-56-treated *POMGnT1*^*-/-*^ RPE cells. Increased phosphorylation of RIP1, RIP3, and MLKL, as well as elevated expression of autophagy markers (p62, Beclin I, and LC3B II/I), was observed in *POMGnT1*^*-/-*^ cells compared with *POMGnT1*^*+/+*^ cells. Treatment with 40 µM RIPA-56 suppressed these alterations. **B** Quantitative analysis of Western blot data normalized to loading control (GAPDH) data. RIPA-56 significantly reduced the phosphorylation of RIP1, RIP3, and MLKL and altered the expression of autophagy markers in *POMGnT1*^*-/-*^ RPE cells. Statistical significance: ns = not significant (*, *p* < 0.05; **, *p* < 0.01; ***, *p* < 0.001; *n* = 12). **C** Schematic representation of the experimental setup for evaluating TEER in RPE monolayers treated with RIPA-56. **D** TEER measurements indicate reduced barrier integrity in *POMGnT1*^*-/-*^ cells compared with that in *POMGnT1*^*+/+*^ cells. RIPA-56 treatment restored TEER values in *POMGnT1*^*-/-*^ cells. (*, *p* < 0.05; **, *p* < 0.01; *n* = 3).

The degeneration of photoreceptors and RPE cells is a hallmark of RP [21]. Enhanced RIP3 immunoreactivity was evident in the RPE layer of 12-month-old *Pomgnt1*^*L120R/L120R*^ mice (Supplementary Fig. 6A, B), providing direct in vivo evidence of necroptosis activation in this compartment. To explore the involvement of the necroptosis pathway in retinal cell death, a *POMGnT1* knockout (*POMGnT1*^*-/-*^) human RPE cell line was established. Immunoblot analysis revealed significant upregulation of the SAG–ENO1 complex proteins in *POMGnT1*^*-/-*^ RPE cells. Furthermore, increased phosphorylation of receptor-interacting protein kinase 1 (RIP1), RIP3, and mixed lineage kinase domain-like protein (MLKL) was observed, along with elevated expression of autophagosome-associated protein markers (Fig. 8A, B). Moreover, transepithelial electrical resistance (TEER), a widely accepted quantitative measure of tight junction integrity in epithelial and endothelial monolayers [22] (Fig. 8C), was used to assess RPE monolayer functionality. TEER values were significantly reduced in *POMGnT1*^*-/-*^ RPE cells, indicating compromised paracellular permeability (Fig. 8D).

RIPA-56 is a highly potent, selective inhibitor of receptor-interacting protein kinase (RIP) with minimal off-target effects and great metabolic stability [23]. Here, it was utilized to inhibit RIP1 expression in *POMGnT1*^*-/-*^ RPE cells. Following treatment with 40 µM RIPA-56 for 24 h, Western blot analysis demonstrated that RIPA-56 significantly decreased RIP1 expression and downregulated key necroptosis protein markers in knockout RPE cells (Fig. 8A, B). Notably, RIPA-56 treatment restored TEER levels, indicating increased barrier integrity in *POMGnT1*^*-/-*^ RPE cells (Fig. 8D). These findings suggest that the loss of POMGnT1 function in RPE cells induces necroptosis through the activation of the RIP1/RIP3/MLKL pathway, contributing to retinal degeneration. The ability of RIPA-56 to mitigate these effects highlights its potential as a therapeutic agent in further in vivo studies targeting necroptosis-mediated retinal cell death.

### AAV8-mediated gene therapy and RIPA-56 administration mitigate retinal degeneration in Pomgnt1^L120R/L120R^ mice

AAV8 has demonstrated exceptional transduction efficiency in photoreceptor cells at intermediate doses, establishing its reliability and efficacy as a vector for therapeutic interventions targeting photoreceptor degeneration [24–28]. To evaluate the therapeutic potential of AAV8-mediated gene delivery and pharmacological intervention in addressing retinal degeneration, the human *POMGnT1* gene was incorporated into an AAV8 vector and subretinally injected into 3-month-old *Pomgnt1*^*L120R/L120R*^ mice. In parallel, the receptor-interacting protein kinase 1 (RIP1) inhibitor RIPA-56 was administered intraperitoneally (IP) to 3-month-old mice. *Pomgnt1*^*L120R/L120R*^ mice.

**Fig. 9:**
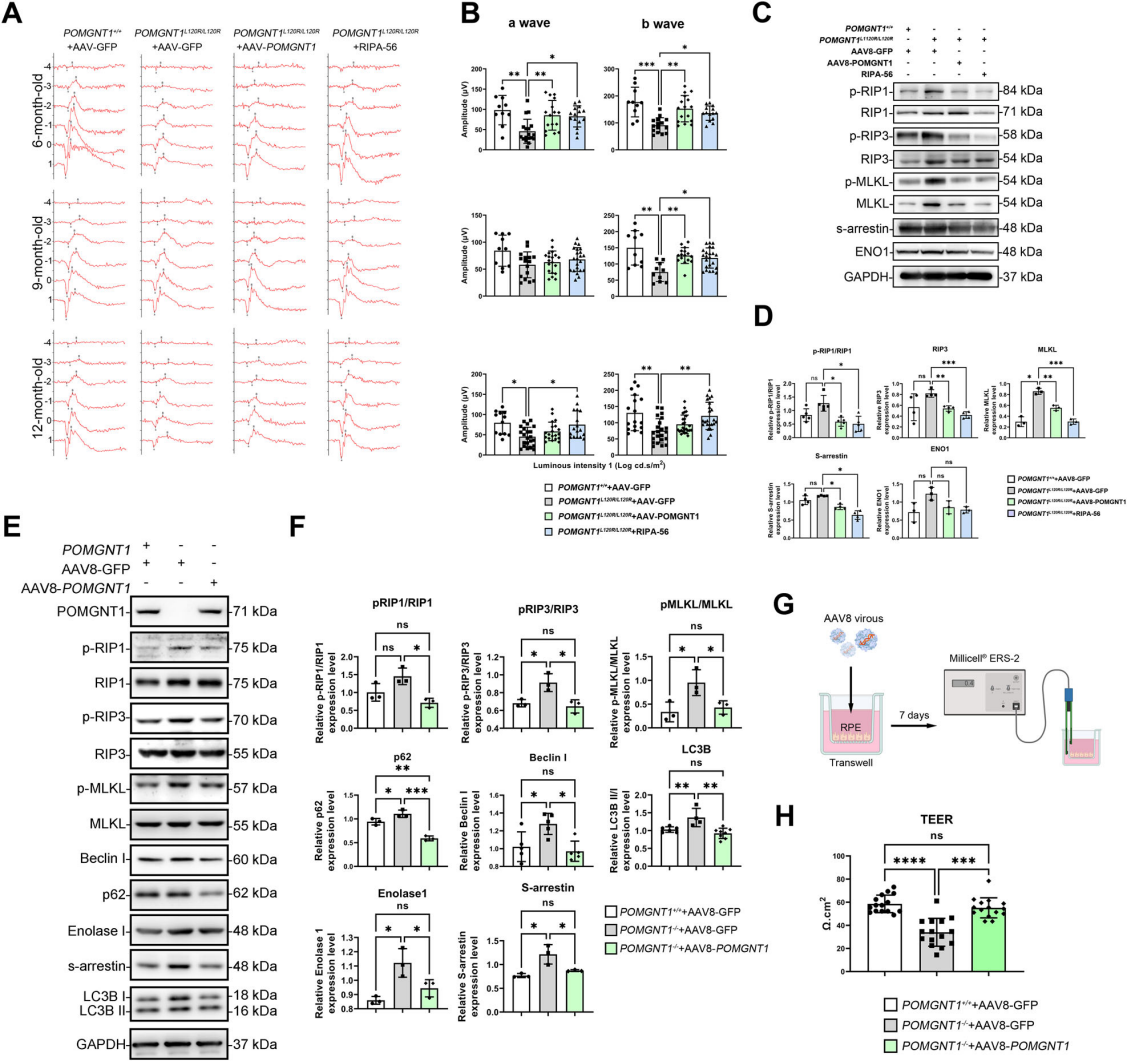
AAV8-mediated h*POMGnT1* gene therapy and RIPA-56 treatment alleviate retinal degeneration and preserve retinal function in *Pomgnt1*^*L120R/L120R*^ mice. **A** Representative ERG recordings from *Pomgnt1*^*+/+*^ (wild-type) mice treated with AAV8-GFP and *Pomgnt1*^*L120R/L120R*^ mice treated with AAV8-GFP, AAV8-h*POMGnT1*, or RIPA-56 at 6, 9, and 12 months of age. **B** Quantification of a- and b-wave amplitudes revealed significantly restored retinal function in AAV8-h*POMGnT1*-treated mice at 6 months and in RIPA-56-treated mice at 6, 9, and 12 months. **C**, **D** Western blot analysis of necroptosis markers (p-RIP1, RIP3, p-MLKL) and photoreceptor/RPE-associated proteins (SAG, ENO1) in the retinas of *Pomgnt1*^L120R/L120R^ mice. Compared with the control, both AAV8-hPOMGnT1 and RIPA-56 reduced the expression of necroptosis-associated proteins. Quantitative analysis of protein expression is shown in (**D**). (*, *p* < 0.05; **, *p* < 0.01; *n* = 12). **E** Western blot analysis of necroptosis and autophagy markers in *POMGnT1*^*+/+*^, untreated *POMGnT1*^−/−^, and AAV8-h*POMGnT1*-transduced *POMGnT1*^−/−^ human RPE cells. AAV8^-^h*POMGnT1* transduction reduced the expression of necroptosis-associated proteins and restored the balance of autophagy markers. **F** Western blot data from (**E**) showed significant reductions in p-RIP1, p-RIP3, and p-MLKL levels in AAV8-h*POMGnT1*-treated cells. (*, *p* < 0.05; **, *p* < 0.01; *n* = 3). **G** Schematic of the Transwell experimental setup for measuring TEER in human RPE cells following AAV8-h*POMGnT1* transduction. **H** TEER measurements indicated greater barrier integrity in *POMGnT1*^−/−^ RPE cells transduced with AAV8-h*POMGnT1* than in control cells. (*, *p* < 0.05; **, *p* < 0.01; ***, *p* < 0.001; ****, *p* < 0.0001; *n* = 3).

ERG was performed at 6, 9, and 12 months of age to assess visual function. The results demonstrated a significant rescue of both a- and b-wave amplitudes in the AAV8-h*POMGnT1*-treated group at 6 months of age. Similarly, RIPA-56 treatment restored a- and b-wave amplitudes in *Pomgnt1*^*L120R/L120R*^ mice by 12 months of age (Fig. 9A, B). Western blot analysis revealed a marked reduction in the expression of the necroptosis-associated proteins RIP1, RIP3, and MLKL in the retinas of 12-month-old *Pomgnt1*^*L120R/L120R*^ mice treated with either AAV8-h*POMGnT1* or RIPA-56 (Fig. 9C, D).

For complementary in vitro studies, human RPE cells deficient in *POMGnT1 (POMGnT1*^*−/−*^*)* were transfected with the AAV8-h*POMGnT1* vector for seven days. TEER measurements demonstrated increased barrier integrity in AAV8-*POMGnT1*-transduced *POMGnT1*^−/−^ RPE cells. Immunoblot analysis revealed a concomitant reduction in RIP1, RIP3, and MLKL protein expression following AAV8-*POMGnT1* transduction (Fig. 9E, F). The functional integrity of the RPE monolayer, as assessed via TEER measurements, indicated reduced paracellular permeability in AAV8-*POMGnT1*-treated *POMGnT1*^−/−^ RPE cells (Fig. 9G, H).

The Golgi complex, which is composed of stacked cisternae near the nucleus, undergoes fragmentation into discrete elements during the early stages of neurodegeneration [29–34]. Furthermore, *POMGnT1* deficiency has been associated with Golgi fragmentation [35], given the colocalization of the protein with the Golgi complex, which serves as a critical hub for protein glycosylation and trafficking. Consistent with these findings, Golgi fragmentation was observed in the *POMGnT1*^−/−^ RPE cells. Treatment with AAV8-*POMGnT1* for seven days attenuated Golgi fragmentation in these cells (Supplementary Fig. 4).

Collectively, these findings suggest that early intervention with AAV8-*POMGnT1* and RIPA-56 in *Pomgnt1*^*L120R/L120R*^ mice mitigates visual impairment by restoring retinal function and inhibiting necroptosis. Additionally, the restoration of Golgi integrity underscores the therapeutic potential of AAV8-*POMGnT1* in addressing the cellular dysfunction associated with *POMGnT1* deficiency.

### AAV8-hPOMGnT1 and RIPA-56 rescue ATP production in POMGnT1-knockout hRPE cells

To evaluate metabolic restoration and cell viability, *POMGnT1*-knockout (KO) human retinal pigment epithelial (hRPE) cells were treated with AAV8-GFP (control), AAV8-*hPOMGnT1*, or 40 µM RIPA-56. ATP concentrations were quantified and expressed as a percentage of wild-type levels (Supplementary Fig. 5A, B). Both treatments significantly improved cellular energy status compared with KO controls. ATP levels increased to 85.6 ± 6.9% following AAV8-*hPOMGnT1* transduction and to 103.7 ± 6.1% after RIPA-56 treatment, relative to 66.4 ± 6.6% and 80.6 ± 6.7% in their respective KO controls. These results indicate that AAV8-mediated *POMGnT1* augmentation and RIPK1 inhibition each effectively rescue bioenergetic function and cell viability in POMGnT1-deficient hRPE cells.

## Discussion

The findings presented in this study reveal the molecular mechanisms underlying retinal degeneration in the *Pomgnt1*^*L120R/L120R*^ mouse model, a condition that mirrors the phenotypic traits observed in patients with homozygous *POMGnT1* L120R mutations. Our results underscore the multifaceted interplay of photoreceptor dysfunction, mitochondrial abnormalities, gliosis, disrupted protein interactions, and necroptosis in driving the pathophysiology of this model while also highlighting potential therapeutic interventions.

Scotopic electroretinography (ERG) analysis revealed a progressive decline in the a- and b-wave amplitudes in *Pomgnt1*^*L120R/L120R*^ mice, which was indicative of significant rod photoreceptor dysfunction and impaired rod-to-rod bipolar cell synaptic transmission. These findings align with the clinical observations of patients harboring homozygous *POMGnT1* L120R mutations who exhibit reduced or absent scotopic ERG responses without associated neuromuscular symptoms [5]. The age-dependent nature of this dysfunction suggests that cumulative photoreceptor damage and synaptic transmission deficits are critical drivers of visual impairment in this model.

Transmission electron microscopy (TEM) and histological analyses revealed marked structural abnormalities in the *Pomgnt1*^*L120R/L120R*^ mouse retina. By 12 months of age, nuclear chromatin dissolution, vacuolization of cytoplasmic organelles, disrupted mitochondrial architecture, and outer nuclear layer (ONL) thinning were evident. These alterations highlight severe retinal degeneration and mitochondrial dysfunction, which are likely key contributors to the observed visual deficits. Mitochondrial dysfunction has been implicated in other retinal degenerative diseases, suggesting a conserved role in photoreceptor pathology [36–43]. The progressive thinning of the ONL underscores the degenerative impact on photoreceptors, which is associated with the functional impairments observed in the ERG.

The upregulation of glial fibrillary acidic protein (GFAP) and glutamine synthetase (GS) in *Pomgnt1*^*L120R/L120R*^ mouse retinas demonstrates a robust gliosis response, which was indicative of neuroinflammation [44–46]. Extending GFAP-positive astrocytic processes to the inner nuclear layer (INL) and outer nuclear layer (ONL) suggests widespread retinal remodeling. While GS upregulation is a compensatory response to glutamate toxicity, excessive GS expression may exacerbate neuronal hyperexcitability and astrocyte swelling [47–50]. These findings suggest that reactive gliosis driven by metabolic and inflammatory stress is a key feature of retinal pathology in POMGnT1-deficient mice.

The observed alterations in retinal glycoprotein profiles, particularly the significantly decreased glycosylation of enolase-1 (ENO1) and α-dystroglycan (α-DG) in the *Pomgnt1*^*L120R/L120R*^ mutant retina, are central to understanding the pathological mechanisms. POMGnT1 is critical for O-mannosyl glycosylation, a post-translational modification essential for the structural integrity and function of numerous glycoproteins, including α-DG, which plays a vital role in maintaining retinal structure and photoreceptor outer segment integrity [51–54]. The reduced glycosylation of α-DG directly correlates with its diminished presence, as shown in Fig. 5A, and likely contributes to the observed retinal thinning, ultrastructural abnormalities, and compromised RPE barrier integrity. This structural compromise would predispose the retina to degeneration.

Furthermore, immunoprecipitation assays revealed that the *POMGnT1* L120R mutation aberrantly enhances the interaction between *POMGnT1* and the SAG–ENO1 complex. S-Arrestin (SAG), also known as S-antigen visual arrestin, plays a critical role in rhodopsin desensitization during phototransduction and participates in light-induced photoreceptor apoptosis through clathrin-mediated endocytosis. Additionally, SAG has been identified as an autoantigen associated with immune responses in uveitis [55–58]. Enolase 1 (ENO1), a glycolytic enzyme critical for cellular energy metabolism, interacts with SAG, and this interaction reduces the catalytic activity of ENO1 by approximately 24% [59]. Given the high energy demand of photoreceptors, with an ATP consumption rate of 10⁸ ATP/s/cell under dark-adapted conditions [60], the reduced glycolytic efficiency caused by increased stabilization of the SAG–ENO1 complex could profoundly affect photoreceptor function.

Glycosylation of ENO1 enhances its enzymatic activity by promoting dimer formation, thereby facilitating aerobic glycolysis; conversely, impaired glycosylation suppresses glycolytic function [61]. The convergence of altered glycoprotein profiles, particularly the hypoglycosylation of ENO1, and enhanced interaction within the SAG–ENO1 complex, result in markedly reduced glycolytic efficiency and subsequent severe ATP depletion in photoreceptors. This compromised energetic state then acts as a critical trigger for the downstream necroptotic pathway, as evidenced by the significant upregulation of RIP1, RIP3 and MLKL. These findings suggest that the *Pomgnt1* L120R mutation stabilizes the SAG–ENO1 complex, exacerbating glycolytic dysfunction and triggering retinal inflammatory responses. The resulting energy deprivation and inflammation likely activate the necroptotic pathway, as indicated by the significant upregulation of the RIP3 and MLKL proteins. The levels of RIP3, a key regulator of necroptosis [62–64], and MLKL, its central downstream effector, were markedly elevated in *Pomgnt1*^*L120R/L120R*^ mouse retinas compared to wild-type mouse retinas. Immunohistochemistry and immunoblotting further supported the role of necroptosis-mediated signaling as a major contributor to retinal neuronal death in this model. These findings highlight the interplay between altered SAG–ENO1 interactions, disrupted energy metabolism, inflammation, and necroptosis as a critical driver of retinal degeneration in *Pomgnt1*^*L120R/L120R*^ mice. Furthermore, these results emphasize the critical role of POMGnT1 in maintaining photoreceptor energy metabolism and cellular homeostasis.

Our findings implicate necroptosis rather than apoptosis as the predominant pathway mediating retinal degeneration in *Pomgnt1*^*L120R/L120R*^ mice. The upregulation of necroptotic markers (RIP1, RIP3, and MLKL) and autophagy-related proteins (Beclin1, p62, and LC3B) highlights the complex interplay between these pathways in retinal cell death. The inhibition of autophagic flux, suggested by elevated p62 levels, may further contribute to cellular dysfunction [20, 65–67]. These data align with emerging evidence that necroptosis is a key driver of neurodegeneration in retinal diseases [68–73].

RIPA-56, a highly potent and selective RIPK1 inhibitor, has substantial therapeutic potential because it attenuates necroptosis, inflammation, ferroptosis, and apoptosis [23, 74, 75]. In a glutamate-induced excitotoxicity model of glaucoma, RIPK1 mitigated retinal ganglion cell damage, demonstrating its neuroprotective efficacy [76]. In addition to its therapeutic effects in ocular models, RIPA-56 effectively reduces organ damage in various inflammatory and degenerative conditions, including diabetic nephropathy [77], acute respiratory distress syndrome [78], and systemic inflammatory response syndrome [23], making it a versatile therapeutic candidate. In *Pomgnt1*^*L120R/L120R*^ mice, pharmacological inhibition of necroptosis with RIPA-56 significantly decreased the expression of necroptotic markers, restored paracellular permeability, and preserved visual function. Similarly, AAV8-mediated h*POMGnT1* gene delivery suppressed necroptosis, improved photoreceptor, and retinal pigment epithelium (RPE) function, and restored Golgi integrity. These therapeutic strategies effectively address the molecular and cellular abnormalities associated with POMGnT1 deficiency. The restoration of Golgi integrity and improved RPE barrier functionality further highlight the therapeutic potential of these approaches. These findings collectively suggest that early intervention targeting necroptosis and metabolic dysfunction may mitigate retinal degeneration and preserve visual function, providing a strong foundation for novel therapeutic strategies.

**Fig. 10:**
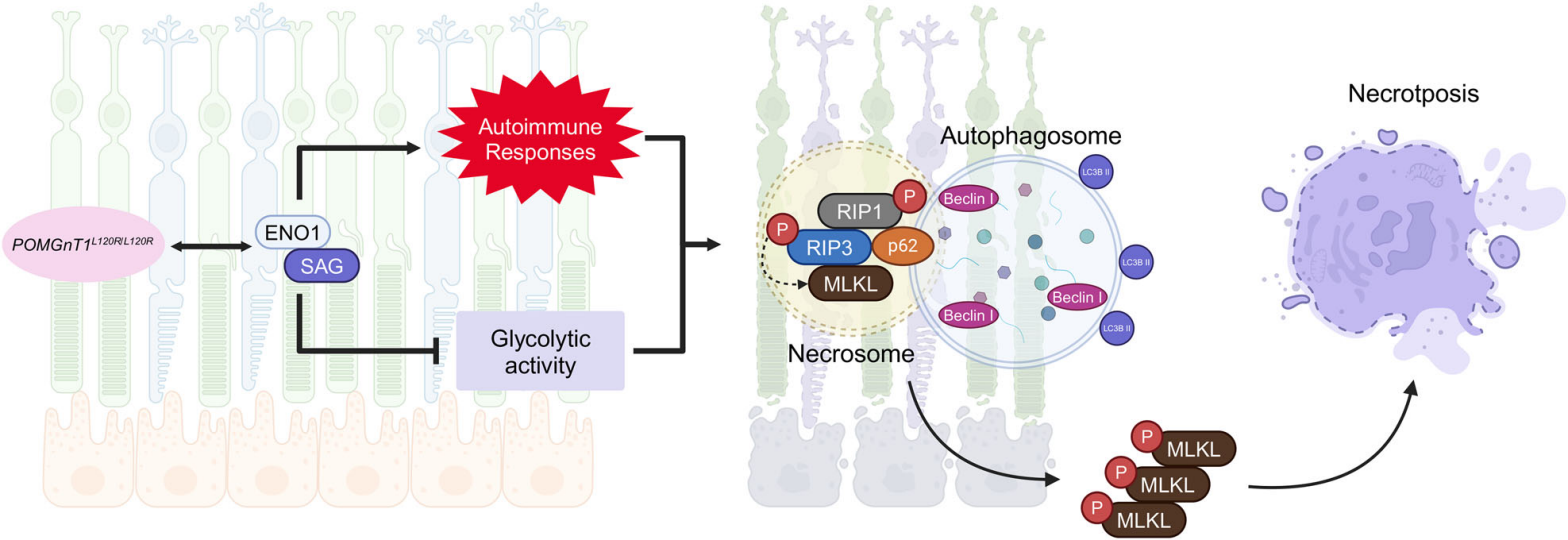
POMGNT1 deficiency induces retinal degeneration via RIPK3-mediated necroptosis and autophagic dysregulation. This schematic illustrates the molecular and cellular pathways involved in retinal degeneration caused by the *POMGNT1* L120R mutation. Mutation of *POMGNT1* triggers aberrant activation of ENO1 and SAG, leading to reduced glycolytic activity and the induction of autoimmune responses. These pathological processes lead to the formation of necrosomes, comprising phosphorylated RIP1 (pRIP1), phosphorylated RIP3 (pRIP3), and MLKL, which mediate necroptotic cell death. Moreover, impaired autophagic flux is evident, characterized by the accumulation of autophagosomes enriched with Beclin I and LC3B-II, reflecting disrupted autophagic homeostasis. The interplay between necroptosis and defective autophagy exacerbates damage to photoreceptor and RPE cells, culminating in retinal degeneration. These findings reveal a novel pathogenic mechanism linking POMGNT1 deficiency to retinal pathology.

In this study, we elucidate the molecular mechanisms underlying POMGnT1-associated retinal degeneration, establishing necroptosis and metabolic dysfunction as central drivers of disease pathogenesis. Our findings reveal that aberrant protein glycosylation—evidenced by significant hypoglycosylation of key metabolic enzymes such as ENO1—precipitates profound metabolic instability and increases the vulnerability of retinal cells. These glycosylation defects disrupt glycolytic capacity, compromise Golgi integrity, deplete cellular energy reserves, and sensitize photoreceptors and RPE cells to RIP1/RIP3-mediated necroptosis (Fig. 10). The preclinical validation of both AAV8-mediated *POMGnT1* gene augmentation and pharmacological RIP1 inhibition with RIPA-56 demonstrates their therapeutic promise, as both approaches effectively attenuated necroptotic signaling, maintained Golgi structure, preserved retinal architecture, and improved visual function. Together, these results provide a compelling rationale for early therapeutic intervention to prevent irreversible retinal degeneration in glycosylation-related diseases. Further studies should focus on optimizing therapeutic delivery, dissecting the complex interplay between necroptosis and autophagy, and broadening the investigation of POMGnT1-related pathways in retinal biology. Collectively, our work highlights the translational potential of targeting glycosylation pathways as a foundation for developing innovative therapies for retinal diseases driven by glycosylation defects.

## Materials and Methods

### Animals

CRISPR/Cas9 genome editing was used to generate *Pomgnt1* L120R mutant and knockout mice, which were obtained from the Transgenic Core Facility (Institute of Molecular Biology, Academia Sinica, Taiwan). The mice were maintained on a C57BL/6JNarl background. The sequence of the L120R single guide RNA (sgRNA) used to target exon 4 in *Pomgnt1* was 5’-TGCCGCTGTGTAGGTGCTCG-3.’ To generate the mutant site via homology-directed repair (HDR), two single-stranded oligodeoxynucleotide (ssODN) constructs harboring the p.L120R (c.359 T > G) mutation and silent nucleotide mutations were designed to prevent Cas9 editing. The sequences of the two ssODNs were 5’-CTATGACATGGATGCCTCGGCCCTGCTCCCGGGCTTCATCCTCGCGCACCTACACAGCGGCAGAAACAGAATCCAGCTATTCACCCTGGCGCTCACGGTGACTCCATCTTAGAAGCAGGAACCACAGCAGCC-3’ and 5’-CGGGCTCTCCCATCCTTAAGCTCACCGTGGCCTGGTTGAGGACTATGACATGGATGCCTCGGCCCTGCTCCCGGGCTTCATCCTCGCGCACCTACACAGCGGCAGAAACAGAATCCAGCTATTCACCCTGGC-3.’ For the POMGNT1 knockout mouse, the sequence of the sgRNA used to target exon 3 in *Pomgnt1* was as follows: 5’-CACTCGGAGAGCAATCAGCG-3.’ To generate the mutant site via homology-directed repair (HDR), a ssODN construct harboring the p.R63* (c.187 A > T) mutation and silent nucleotide mutations was designed to prevent Cas9 editing. The sequences of the two ssODNs were as follows: 5’-GGTCCCGTCCCGTCCCCCGCCCCCCTCCCGCACCCTCAGCTGGTCCCCACCATAGTCTTGCTCTGGTTCTGGGTCTTCATTGGCCTCGCTGATTGCTCaCCGAGTGTCCAGGATCAACTTGATGTTGACAATGAC-3’. ssODNs and sgRNA were mixed with Cas9 RNA and microinjected into the pronuclei of zygotes. Zygotes were transplanted into the oviducts of pseudopregnant female ICR mice (Supplementary Fig. 1A). C57BL/6JNarl wild-type (WT) mice were purchased from the National Laboratory Animal Center of Taiwan. All experimental procedures involving animals were performed in strict accordance with the ARVO Statement for the Use of Animals in Ophthalmic and Vision Research and the Guide for the Care and Use of Laboratory Animals. The experimental protocol was approved by the Institutional Animal Care and Use Committee (IACUC) of Tzu Chi University (Approval No.: 109026 (06/08/2020) and 10nts adhered to ARRIVE guidelines for reporting animal research.

CRISPR/Cas9 genome editing was used to generate *Pomgnt1* L120R mutant and knockout mice, which were obtained from the Transgenic Core Facility (Institute of Molecular Biology, Academia Sinica, Taiwan). The mice were maintained on a C57BL/6JNarl background. The sequence of the L120R single guide RNA (sgRNA) used to target exon 4 in *Pomgnt1* was 5’-TGCCGCTGTGTAGGTGCTCG-3.’ To generate the mutant site via homology-directed repair (HDR), two single-stranded oligodeoxynucleotide (ssODN) constructs harboring the p.L120R (c.359 T > G) mutation and silent nucleotide mutations were designed to prevent Cas9 editing. The sequences of the two ssODNs were 5’-CTATGACATGGATGCCTCGGCCCTGCTCCCGGGCTTCATCCTCGCGCACCTACACAGCGGCAGAAACAGAATCCAGCTATTCACCCTGGCGCTCACGGTGACTCCATCTTAGAAGCAGGAACCACAGCAGCC-3’ and 5’-CGGGCTCTCCCATCCTTAAGCTCACCGTGGCCTGGTTGAGGACTATGACATGGATGCCTCGGCCCTGCTCCCGGGCTTCATCCTCGCGCACCTACACAGCGGCAGAAACAGAATCCAGCTATTCACCCTGGC-3.’ For the POMGNT1 knockout mouse, the sequence of the sgRNA used to target exon 3 in *Pomgnt1* was as follows: 5’-CACTCGGAGAGCAATCAGCG-3.’ To generate the mutant site via homology-directed repair (HDR), a ssODN construct harboring the p.R63* (c.187 A > T) mutation and silent nucleotide mutations was designed to prevent Cas9 editing. The sequences of the two ssODNs were as follows: 5’-GGTCCCGTCCCGTCCCCCGCCCCCCTCCCGCACCCTCAGCTGGTCCCCACCATAGTCTTGCTCTGGTTCTGGGTCTTCATTGGCCTCGCTGATTGCTCaCCGAGTGTCCAGGATCAACTTGATGTTGACAATGAC-3’. ssODNs and sgRNA were mixed with Cas9 RNA and microinjected into the pronuclei of zygotes. Zygotes were transplanted into the oviducts of pseudopregnant female ICR mice (Supplementary Fig. 1A). C57BL/6JNarl wild-type (WT) mice were purchased from the National Laboratory Animal Center of Taiwan. The study protocol was approved by the Institutional Animal Care and Use Committee (IACUC) of Tzu Chi University (Approval No.: 109026 (06/08/2020) and 112057 (04/24/2024)). All experimental procedures involving animals were performed in strict accordance with the ARVO Statement for the Use of Animals in Ophthalmic and Vision Research and the Guide for the Care and Use of Laboratory Animals. Animals were housed under controlled environmental conditions (12-hour light/dark cycle, temperature 22 °C ± 2 °C, humidity 50–60%) with ad libitum access to food and water at the Laboratory Animal Center, Tzu Chi University.

### Genotyping

In all the experiments, the genomic DNA was subjected to PCR to confirm that the mice contained the p.L120R mutant site. The toes were collected from 10-day-old mice and homogenized using DirectPCR™ lysis reagent (Cat# 101-T; Viagen Biotech, US) supplemented with 50 mg/kg proteinase K (Cat# 17916; Thermo, USA). A 432-bp pomgnt1 gene was amplified using the amaR OnePCR™ kit (Cat#SM216-0250; GeneDireX, Taiwan) using modified forward (5’-CGGGCAGCTAACTCCCTTAT-3’) and reverse (5’-GGCCTGGTTGAGGACTATGA-3’) primers. For enzymatic digestion of the pomgnt1 gene c.359 T > G (p.L120R), 432-bp PCR products were cleaved into fragments of 374 and 58 bp by the Bsh1236I enzyme (BstUI, Cat# ER0921, Thermo, US). The genotypes were *Pomgnt1*^*+/+*^ (432 bp), *Pomgnt1*^*L120R/+*^ (432 bps+374 bps+58 bps), and *Pomgnt1*^*L120R/L120R*^ (374 bps+58 bps) (Supplementary Fig. 1B).

### ERG

ERG was used to assess the retinal function of 6-, 9-, and 12-month-old WT and *Pomgnt1*^L120R/L120R^ mice. After 12 h of dark adaptation, the mice were anesthetized with a mixture of ketamine and xylazine (100 mg/kg; 10 mg/kg body weight). Eyes were mixed with Mydrin-P (Santen, Osaka, Japan) and 0.5% Alcaine (Alcon, Puurs, Belgium) for all procedures. The mice were placed on the platform to maintain their body temperature at 37 °C. Two electrodes were attached to the eyes, and flash ERG data were recorded using the CELERIS system (Diagnosys, US). ERG responses were measured under dark-adapted conditions (light intensities of 0.0001, 0.001, 0.01, 0.1, 1, and 10 cd-s/m^2^).

### Protein isolation for mass spectrometry analysis

The retinas were dissected from 6-month-old mice, and the tissues were lysed with RIPA buffer. The proteins were separated via 8% SDS‒PAGE and visualized using iBlue protein stain solution (SJ003-1000 M, GeneDireX, USA). The excised bands were digested overnight with trypsin at 37 °C. The supernatant was collected and analyzed by liquid chromatography‒tandem mass spectrometry (Orbitrap, Thermo, US) (services provided by the Metabolomics‒Proteomics Research Center of National Yang Ming Chiao Tung University).

### AAV viral vector

AAV8-h*POMGnT1*-eGFP (AA08-H4600-AV08-400, GeneCopoeia, USA) and control AAV8-eGFP (AA08-EGFP-AV02-025, GeneCopoeia, USA) were used in all the experiments. The vector with human *POMGnT1* (NM_017739.4) mRNA, which was 1983 bp, was packaged into an AAV8 capsid and harbored an eGFP expression cassette driven by the cytomegalovirus (CMV) promoter.

### AAV8 subretinal injection and RIPA-56 administration

Three-month-old mice were anesthetized with a mixture of ketamine and xylazine (100 mg/kg; 10 mg/kg body weight). Eye drops consisting of Mydrin-P (Santen, Osaka, Japan) and 0.5% Alcaine (Alcon, Puurs, Belgium) were used for all procedures. The eye was punctured with a 31 G needle (Terumo, Japan), and AAV8-h*POMGnT1*-eGFP (~5 × 10^9^ viral genomes/μl) and control AAV8-eGFP (~5 × 10^9^ viral genomes/μl) were subretinally injected using a NanoFil syringe and 34 G blunt needle (World Precision Instruments, Sarasota, FL, USA). RIPA-56 (HY-101032, MedChemExpress, US) was diluted in 40% PEG300 solution (HY-Y0873, MedChemExpress, US) and intraperitoneally injected into 3-month-old mice at 10 mg/kg twice a week for 3 and 9 months.

### Coimmunoprecipitation

After euthanasia via CO_2_ inhalation, retinas from the mice were homogenized in RIPA buffer (50 mM Tris-HCl pH 7.6, 150 mM NaCl, 1 mM EDTA, and 1% NP-40). The proteins were sonicated for 30 s on ice and centrifuged at 14,000 × g for 15 min. Equal to the supernatant, the concentrations were confirmed with a BSA protein assay kit (Cat#23225, Thermo, US). To bind the antibodies to Dynabeads™ Protein G (1.5 mg, 10003D, Thermo, US), the anti-enolase1 (10 μg, ab155102, Abcam, US), anti-s-arrestin (10 μg, NBP2-25161, Novus, US), and anti-Pomgnt1 (10 μg, WH0055624M7, Sigma, US) antibodies were incubated with Dynabeads™ Protein G (10003D, Thermo, US) at 4 °C overnight on a shaker plate at 50 rpm, and 1 mg of retinal protein was incubated with Dynabeads™ Protein G at 4 °C overnight on a shaker plate at 50 rpm. Mouse control IgG (10 μg, AC011, ABclonal, US) was used as the nonspecific antibody control. The protein was eluted, and the pellets were washed three times with elution buffer. Immunoprecipitated proteins were separated by 10% SDS‒PAGE and transferred to a polyvinylidene fluoride (PVDF) membrane. The results of the blot analysis were confirmed with three targeted proteins and photographed using a ChemiDoc™ MP Imaging System (Bio-Rad, US).

### Glycoprotein isolation from retina

Glycoproteins were isolated from retinal tissues of wild-type (Pomgnt1^+/+^) and mutant (Pomgnt1^L120R/L120R^) mice using a Pierce™ Glycoprotein Isolation Kit (Wheat Germ Agglutinin, WGA) (#89805, Thermo Fisher Scientific Inc, MA, USA) according to the manufacturer’s instructions. Briefly, retinas were dissected, homogenized in lysis buffer provided in the kit, and incubated on ice for 30 min. Lysates were centrifuged at 14,000 × g for 15 min, and the supernatants were collected. Protein concentrations were quantified using a BCA protein assay. Equal amounts of total protein (500 µg) were applied to lectin columns provided in the kit to selectively bind glycoproteins. Columns were washed to remove unbound proteins, and glycoproteins were eluted using elution buffer. The isolated glycoproteins were then analyzed by Western blotting to evaluate differences in glycoprotein profiles between wild-type and mutant retinas. Alpha-dystroglycan (α-DG), a known substrate of POMGnT1-dependent O-mannosyl glycosylation [79, 80], was used as a positive control to confirm the specificity and effectiveness of the glycoprotein isolation and detection protocol. In contrast, beta-dystroglycan (β-DG), which does not undergo O-mannosyl glycosylation, was included as a negative control in the WGA pulldown assay.

### Immunohistochemistry

After euthanasia via CO_2_ inhalation, the mouse eyes were dissected and fixed in 4% paraformaldehyde (#43368, Thermo, USA) for 1 h at room temperature. Eyes were dehydrated in 30% sucrose (#107651, Merck, US) and embedded in optimal cutting temperature (OCT) compound (#4583, Sakura, US) at −80 °C. Retinal sections were prepared (10 μm) from the frozen tissue and were washed in PBS and blocked with 1% bovine serum albumin (BSA) containing 1% normal goat serum for 1 h. The sections were incubated with primary antibodies against GFAP (1:200, ab7260, Abcam, US), RIP3 (1:100, NBP1-77299, Novus, US), enolase-1 (1:1000, ab155102, Abcam, US), S-arrestin (1:1000, NBP2-25161, Novus, US), and LC3B (1:100, ab51520, Abcam, US). The sections were then incubated with the corresponding Alexa Fluor-conjugated secondary antibodies (1:100, Invitrogen, US), and counterstaining was performed using DAPI (1:500, #10236276001, Sigma, US). Photographs were obtained using a Zeiss LSM 900 confocal system (Zeiss, Germany). At least six images per eye at 20× magnification were used for retinal quantification.

### Terminal-deoxynucleotidyl-transferase-mediated nick end labeling (TUNEL) assay

After euthanasia via CO_2_ inhalation, the mouse eyes were dissected and fixed with 4% paraformaldehyde (#43368, Thermo, USA) for 1 h at room temperature. Eyes were dehydrated in 30% sucrose (#107651, Merck, US) and embedded in OCT compound (#4583, Sakura, US) at −80°C. The 10 μm-thick retinal frozen sections were subjected to a TUNEL assay (Click-iT™ Plus TUNEL Assay for In Situ Apoptosis Detection, Invitrogen, Waltham, MA, US). Apoptotic cells were detected using a confocal microscope (LSM900, Zeiss). The number of TUNEL-positive cells in the retina was counted in 10 high-power fields (HPFs; 400 × magnification). The average of three sections per mouse was calculated. Positive cells were measured using ImageJ software.

### Cell culture

Immortalized human RPE cells (#T0571, Abcam, US) were cultured in Dulbecco’s modified Eagle’s medium/nutrient mixture F-12 (DMEM/F12, #11330-032, Gibco, US). The medium was supplemented with 10% fetal bovine serum (FBS, #10082147, Gibco, US), 1% L-glutamine (200 mM, #25030081, Gibco, US), and 1% penicillin‒streptomycin (10,000 U/mL, #15140122, Gibco, US), and the cells were incubated in an incubator at 37 °C with 5% CO_2_. The cells were subcultured with 0.05% trypsin-EDTA (#15400054; Gibco, US) every 4‒6 days. RIPA-56 (HY-101032, MedChemExpress, US) and dabrafenib (HY-14660, MedChemExpress, US) were dissolved in DMSO. In the cell viability experiment, the cells were seeded onto 96-well plates (1 × 10^4^ cells/well) and treated with 10–100 µM RIPA-56 and 0.5–10 µM dabrafenib for three days. The WST-1 assay (ab155902, Abcam, US) is a common assay for detecting cell viability that results in formazan formation after the reagent binds to cellular dehydrogenases. After 2 h of incubation, the absorbance was measured at 450 nm using a plate reader. The relative percentage of viable cells in each group was calculated by comparing the absorbance values of the experimental groups with that of the control group.

### Pomgnt1 knockout in the hRPE cell line via CRISPR/Cas9

To generate a *POMGnT1* knockout cell line, a CRISPR/Cas9 system was purchased from the National RNAi Core Facility, Academia Sinica, Taipei, Taiwan. A sgRNA targeting the *POMGnT1* gene, 5’-GCACCCATACTGTGTGACAT-3’, was cloned and inserted into a pAll-Cas9 pPuro lentiviral vector. hRPE cells were transfected with plasmids carrying individual sgRNAs. After the cells were sorted and grown to confluence, the *POMGnT1* knockout efficiency was confirmed by Western blotting.

### TEER measurements

For transport studies, hRPE cells were seeded at a density of 1 × 10^5^ cells/well on Transwell inserts (0.9 cm² of cell growth area, 0.4 µm pore size, #353180, Falcon, US). The *POMGnT1*^*-/-*^ groups were treated with 40 µM RIPA-56 and 2 µM dabrafenib for three days. TEER was measured using a Millicell-ERS2 Voltohmmeter (MERS00002, Millipore, US). The electrode was sterilized in isopropanol and equilibrated in PBS (#10010023, Gibco, US) for 15 min. The electrode was immersed in the Transwell inserts to record the degree of resistance of the cells.

### AAV8 transfection

hRPE cells were seeded at 30–40% confluency in 6-well plates. hRPE cells were incubated with 1% FBS culture medium for 1 h and infected with AAV8-h*POMGnT1*-eGFP or control AAV8-eGFP (multiplicity of infection (MOI) = 10,000; the MOI calculation was based on an estimated number of HeLa cells/12-well plates at 90% confluence (https://www.thermofisher.com/tw/zt/home/references/gibco-cell-culture-basics/cell-culture-protocols/cell-culture-useful-numbers.html) for 1 d. Complete culture medium was then added and incubated for 7 days. The cells were incubated with DAPI (R37606, Thermo, US) for 30 min and then photographed using an EVOS M5000 Imaging System (AMF5000, Thermo, US). For each group, eight to ten representative images were taken from 8 to 10 representative wells. The density of FITC/DAPI double-stained cells and the number of cells were quantified using Celleste™ 6 Image Analysis Software (Thermo, US).

### Immunocytochemistry of the hRPE cell line

The cells were transferred to 4-well chamber slides (seeding density: 4 × 10^5^ cells/well) and cultured for 24 h. The cells were treated with 40 µM RIPA-56 for three days. The cells were fixed in 4% paraformaldehyde (#43368; Thermo Fisher Scientific) for 20 min at room temperature. After the cells were washed in PBS three times, they were incubated with anti-LC3B (1:100, S83506, Cell Signaling, US) and anti-LAMP1 (1:00, ab24170, Abcam, US) primary antibodies at 4 °C overnight. After washing, the cells were incubated with corresponding Alexa Fluor (1:100, Invitrogen, US)-conjugated secondary antibodies at room temperature for 1 h and counterstained with DAPI (1:500, #10236276001, Sigma, US). Photographs were taken with a Zeiss LSM 900 confocal system (Zeiss, Germany). At least six images per eye were captured at 63× magnification for the quantification of cells.

### Immunoblotting

Mouse retinal or hRPE cells were lysed with RIPA buffer (50 mM Tris-HCl pH 7.6, 150 mM NaCl, 1 mM EDTA, and 1% NP-40). Protein concentrations were confirmed using a BSA protein assay kit (#23225, Thermo, USA). Protein samples (30 µg) were separated via 8% SDS‒PAGE and transferred to a PVDF membrane. After 1 h of blocking with 5% nonfat milk, the membranes were incubated with primary antibodies against RIP1 (1:500, #610458, BD, US), RIP3 (1:500, S95702, Cell Signaling, US), MLKL (1:200, PA5-43960, Invitrogen, US), enolase1 (1:10000, ab155102, Abcam, US), s-arrestin (1:10000, NBP2-25161, Novus, US), p62 (1:1000, ab91526, Abcam, US), Beclin I (1:1000, ab62557, Abcam, US), LC3B (1:1000, S83506, Cell Signaling, US), and Pomgnt1 (1:1000, WH0055624M7, Sigma, US) at 4 °C overnight. After being washed, the blots were incubated with appropriate horseradish peroxidase-conjugated secondary antibodies (1:10,000, Bio-Rad, US) at room temperature for 1 h. The protein bands were detected via an enhanced chemiluminescence (ECL) system (GERPN2232, Cytiva, UA) and a ChemiDoc™ MP Imaging System (Bio-Rad, US). An anti-GAPDH antibody (1:10,000, G8795, Sigma, US) was used as an internal loading control. The blot analysis was conducted using Image Lab software (version 6.0.1; Bio-Rad, US).

### TEM of retinas

After euthanasia via CO_2_ inhalation, mouse eyes were prefixed in 2.5% glutaraldehyde and 0.1 M cacodylate buffer with 1% tannic acid. Retinal tissues from 1 mm of the optic nerve disc were removed using a biopsy punch. The samples were subsequently fixed with 1% osmium tetroxide and 0.1 M cacodylate buffer. Following postfixation, the tissues were incubated in 2% uranyl acetate. The retina was then embedded in Spurr’s resin. Next, 80-nm-thick cross-sections were obtained using an ultramicrotome (EM UC6, Leica, Germany) and observed using a transmission electron microscope (H-7500, Hitachi, Japan).

### Histology

After euthanasia via CO_2_ inhalation, the mouse eyes were dissected and fixed with 4% paraformaldehyde (#43368, Thermo, USA) for 1 h at room temperature. Eyes were dehydrated in 30% sucrose (#107651, Merck, US) and embedded in OCT compound (#4583, Sakura, US) at -80°C. Retinal sections (10 μm) were washed with PBS and stained with hematoxylin and eosin (H&E). The retinal sections from three consecutive images were examined at a magnification of 20×, and color images were obtained using light microscopy (AxioScope 5, Zeiss, Germany). Images were analyzed using the ImageJ software. The total retinal thickness and the number of photoreceptor nuclei rows in the outer nuclear layer (ONL) were quantified at 200 μm intervals along the retina, extending from the optic nerve head to the periphery.

### Statistics

The data are presented as the mean ± SDs. Statistical analysis was performed using Prism software (version 8; GraphPad, US). The Kruskal‒Wallis test and Mann‒Whitney U test were used for comparisons between each group. In all cases, *p* < 0.05 was considered statistically significant.

## Supplementary information

Supplementary information

Raw data gel blot

## Data Availability

All data generated or analyzed during this study are included in this published article [and its supplementary information files].
