# Supplementary material for: Glycosylation-driven necroptosis in retinal degeneration: dual rescue by AAV8 gene therapy and RIPK1 inhibition
Source: Cell Death Discov. 2026 Apr 9;12:241. doi: 10.1038/s41420-026-03098-8 (PMC13187256; doi:10.1038/s41420-026-03098-8)
Supplement: Supplementary file 1 — Supplementary information [file 41420_2026_3098_MOESM1_ESM.docx]

Supplementary information

1. Generation of *Pomgnt1^L120R/L120R^* mice and genotyping

A CRISPR/Cas9 genome editing was used to generate the *Pomgnt1^L120R/L120R^* mutant and knockout mice and purchased from the Transgenic Core Facility (Institute of Molecular Biology, Academia Sinica, Taiwan). The mice were maintained on the C57BL/6J background. The sequence of the L120R single guide RNA (sgRNA) used to target exon 4 in *POMGNT1* was follow: 5’-TGCCGCTGTGTAGGTGCTCG-3’. To generate the mutant site via homology-directed repair (HDR), two single-stranded oligodeoxynucleotides (ssODN) construct harboring the p.L120R (c.359T>G) mutation and silent nucleotide mutations to prevent Cas9 editing was designed. The sequence of two ssODN were as follows: 5’-CTATGACATGGATGCCTCGGCCCTGCTCCCGGGCTTCATCCTCGCGCACCTACACAGCGGCAGAAACAGAATCCAGCTATTCACCCTGGCGCTCACGGTGACTCCATCTTAGAAGCAGGAACCACAGCAGCC-3’ and 5’-CGGGCTCTCCCATCCTTAAGCTCACCGTGGCCTGGTTGAGGACTATGACATGGATGCCTCGGCCCTGCTCCCGGGCTTCATCCTCGCGCACCTACACAGCGGCAGAAACAGAATCCAGCTATTCACCCTGGC-3’. The ssODN and sgRNA were mixed with Cas9 RNA and microinjected into pronuclei of zygotes. The zygotes were transplanted into the oviducts of pseudo pregnant female ICR mice. The genomic DNA was using PCR to conform mice containing p.L120R mutant site. The toes were collected form mouse pups over 10-day-old using DirectPCR™ lysis reagent (Cat#101-T, Viagen Biotech, US) with 50 mg/kg Proteinase K (Cat#17916, Thermo, USA). A 432-bp length of the *pomgnt1* gene was amplified using amaR OnePCR™ kit (Cat#SM216-0250, GeneDireX, US) and modified from forward (5’-CGGGCAGCTAACTCCCTTAT-3’) and reverse (5’-GGCCTGGTTGAGGACTATGA-3’) primers. For enzymatic digestion of *pomgnt1* gene c.359T>G (p.L120R), the 432-bp of PCR products were fragmented into fragments of 374 and 58 bps by Bsh1236I enzyme (BstUI, Cat# ER0921, Thermo, US). The genotype as follows: *Pomgnt1^+/+^* (432 bps), *Pomgnt1^L120R/+^* (432 bps+374 bps+58 bps), and *Pomgnt1^L120R/L120R^* (374 bps+58 bps) (Supplementary figure 1).


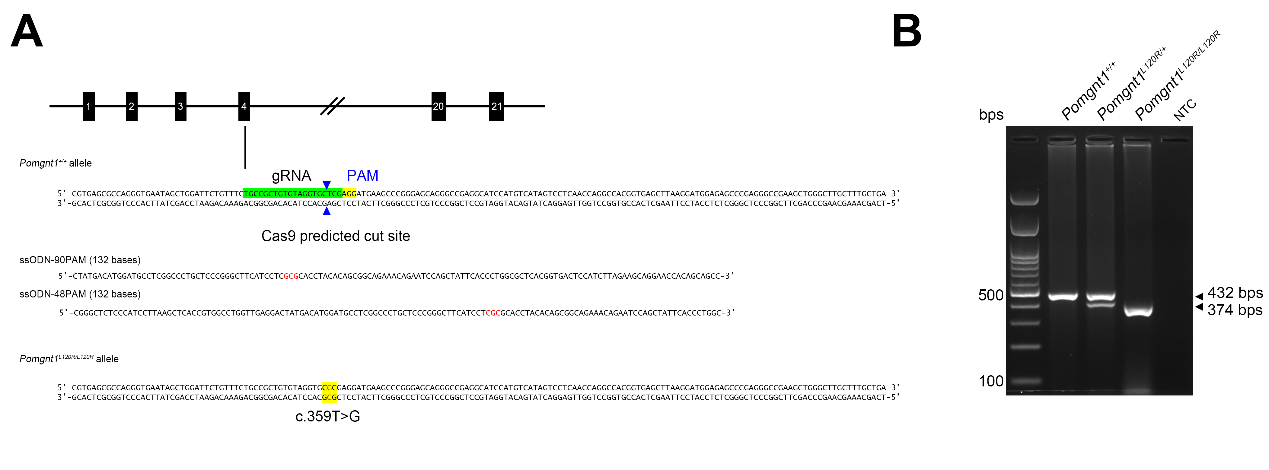
 (Supplementary figure 1)

1. A representative view of the CRISPR/Cas9 targeting strategy used for generating *Pomgnt1^L120R/L120R^* mice.
2. A representative PCR genotyping result for *Pomgnt1* WT, heterozygous (Het) *Pomgnt1^L120R/+^*, homozygous (*Pomgnt1^L120R/L120R^*) mice.
3. Histology

After euthanasia with CO_2_, mouse eyes were dissected and fixed at 4% paraformaldehyde (#43368, Thermo, US) for 1 hour at room temperature. Eyes were dehydrated at 30% sucrose (#107651, Merck, US) and embedded with O.C.T compound (#4583, Sakura, US) at -80℃. Retinal slides from frozen section with 10 μm were washed in PBS and stained with hematoxylin and eosin (H&E). Retinal sections from 3 consecutive images were examined at 20× magnification by light microscopy (AxioScope 5, Zeiss, Germany), and color images were examined by light microscopy (AxioScope 5, Zeiss, Germany). Images were analyzed using ImageJ software. The total retinal thickness and the number of photoreceptor nuclei rows in the outer nuclear layer (ONL) were quantified at 200 μm intervals along the retina, extending from the optic nerve head to the periphery.


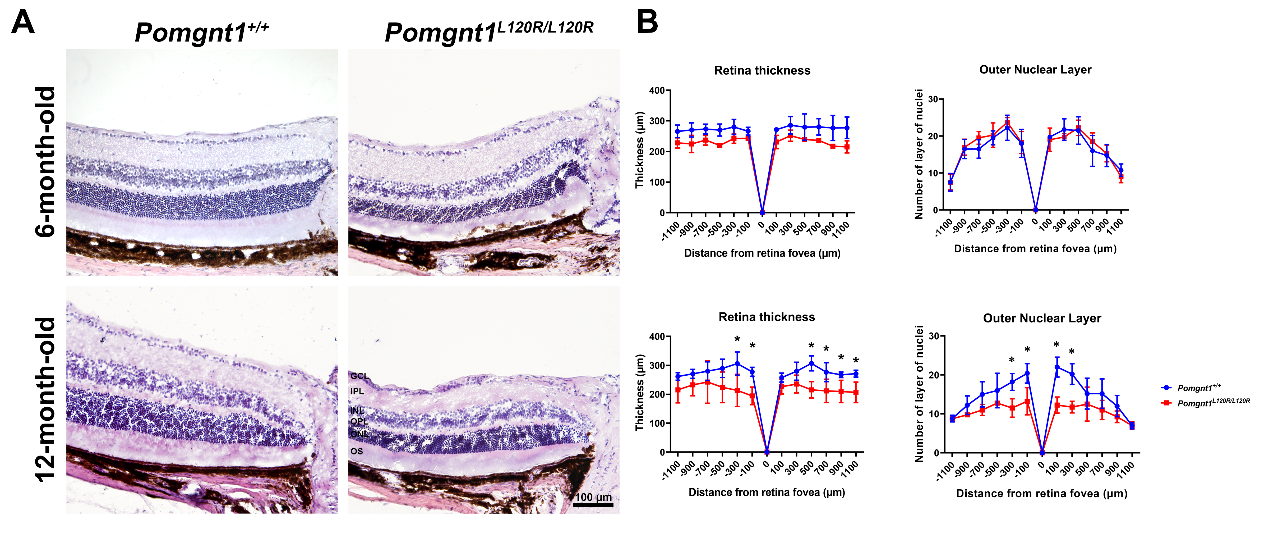


(Supplementary figure 2)

1. Retinal histology in *Pomgnt1^+/+^* and *Pomgnt1^L120R/L120R^* mice at 6- and 12-month-old mice. (GCL: Ganglion Cell Layer; IPL: Inner Plexiform Layer; INL: Inner Nuclear Layer; OPL: Outer Plexiform Layer; ONL: Outer Nuclear Layer; OS: Outer Segments; Scale bars: 100 μm.)
2. Quantitative analysis of the total retinal thickness and the number of photoreceptor nuclei rows in the outer nuclear layer (ONL) of *Pomgnt1^+/+^* and *Pomgnt1^L120R/L120R^* mice at 6 and 12 months of age. (*, p<0.05; n=12)
3. Terminal-Deoxynucleotidyl-Transferase-Mediated Nick End Labeling (TUNEL) assay

After euthanasia with CO_2_, mouse eyes were dissected and fixed at 4% paraformaldehyde (#43368, Thermo, US) for 1 hour at room temperature. Eyes were dehydrated at 30% sucrose (#107651, Merck, US) and embedded with O.C.T compound (#4583, Sakura, US) at -80℃. The 10 μm retinal frozen sections were performed by TUNEL reaction (Click-iT™ Plus TUNEL Assay for In Situ Apoptosis Detection, Invitrogen, Waltham, MA, US). The slides were used to detect apoptotic cells by confocal microscopy (LSM900, Zeiss). The number of TUNEL-positive cells in the retina was counted in 10 high-powered fields (HPFs; 400 × magnification). Three sections per mouse were averaged. The positive cells were measured using ImageJ software (Supplementary figure 2).


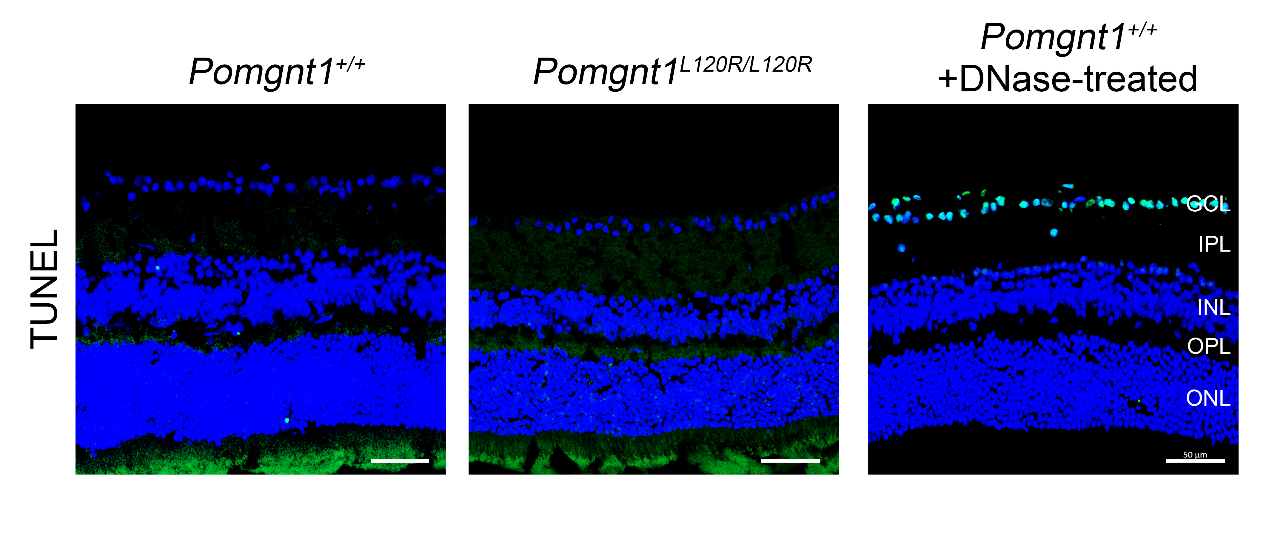


(Supplementary figure 3)

The representative of TUNEL-positive cells in frozen retinal sections of 12-month-old *Pomgnt1^L120R/ L120R^* and *Pomgnt1^+/+^* mice, respectively. TUNEL assay did not detect any positive cells in *Pomgnt1^L120R/ L120R^* and *Pomgnt1^+/+^* mice (scale bar = 50 µm, GCL: Ganglion Cell Layer; IPL: Inner Plexiform Layer; INL: Inner Nuclear Layer; OPL: Outer Plexiform Layer; ONL: Outer Nuclear Layer).

4. Immunocytochemistry (ICC) of hRPE cell line

The cells were transferred to 4-well chamber slide (seeding density: 4×105 cells/well) and cultured for 24 hours. The cells were treated with 40 µM of RIPA-56 for 3 days. The cells were fixed at 4% paraformaldehyde (#43368, Thermo, US) for 20 minutes at room temperature. After washing the cells in PBS for 3 times, the cells were incubated with GM130 (1:250, ab52649, abcam, US) primary antibody at 4℃ overnight. After washing, the cells were incubated with corresponding Alexa Fluor (1:100, Invitrogen, US) conjugated secondary antibodies at room temperature for 1 hour, counterstaining was performed using DAPI (1:500, #10236276001, Sigma, US). Photographs were taken by the Zeiss LSM 900 confocal system (Zeiss, Germany). At least six images per eye by 63× magnification were taken for the quantification of cells.


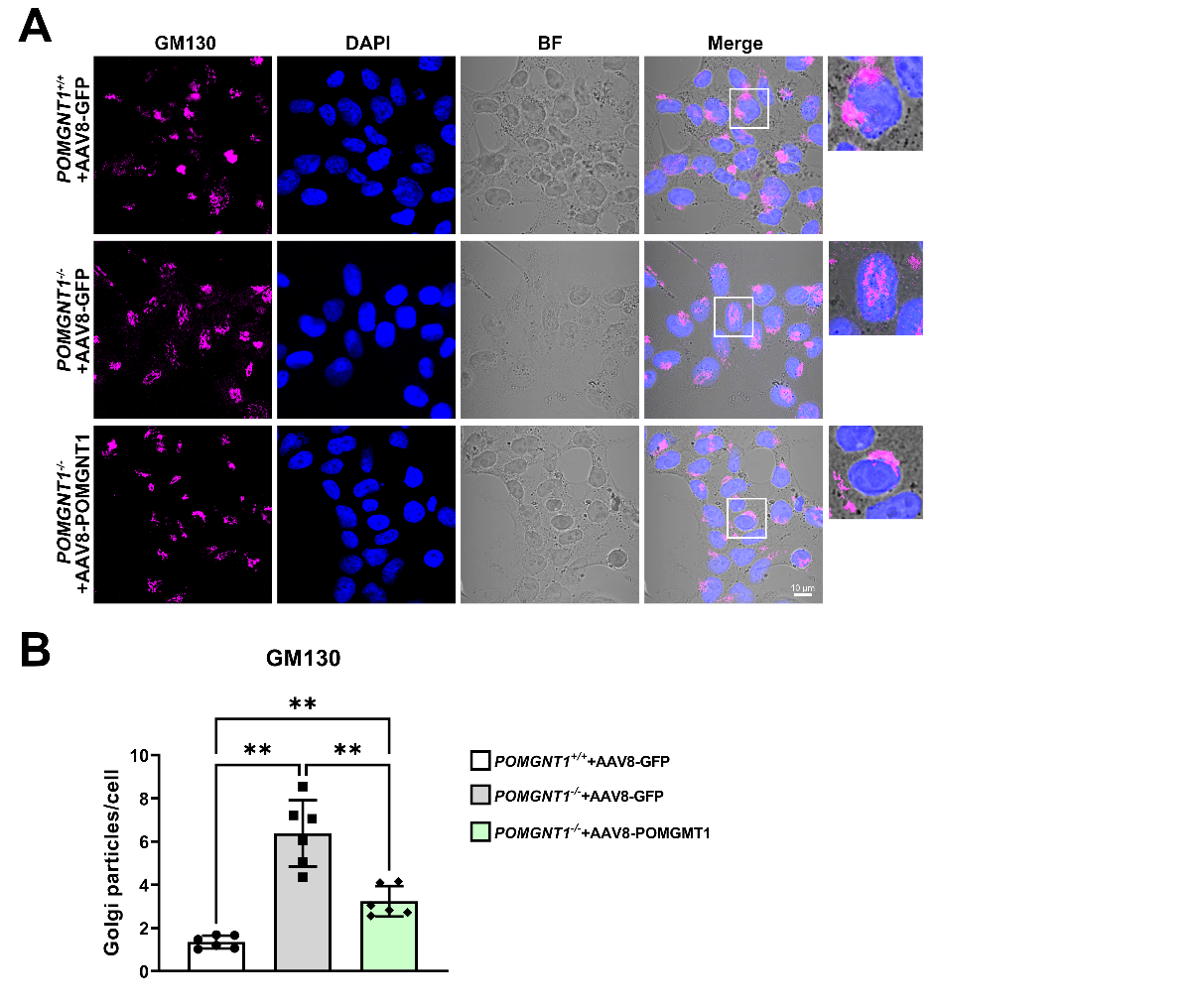


(Supplementary figure 4)

1. Representative images of GM130-positive cells after treatment with AAV8-*POMGnT1* for 7 days. Cells were immunostained with GM130-specific antibodies (A, purple) and DAPI (A, blue).
2. Quantification shows a significant decrease in GM130-positive particles in the AAV8-*POMGnT1* treatment group compared to the untreated group (*, p < 0.05; **, p < 0.01; n = 6).

5. *POMGnT1* gene augmentation and RIPK1 inhibition restore ATP production in POMGnT1 knockout cells.

hRPE was grown on the 96-well plate in culture medium. Cells were treated AAV8-GFP, AAV8-*POMGnT1*, and 40 μM RIPA-56, respectively. The Luminescent ATP Detection Assay Kit (ab113849, Abcam, US) was employed to measure cell viability. The luminescence values were converted to ATP concentrations using a standard curve. The cells were then mixed with Substrate Solution for 5 minutes, shaking at 600 rpm. The plate was incubated while covered for 10 minutes. Luminescence was measured using a GloMix® Discover Microplate Reader (Promega, US).


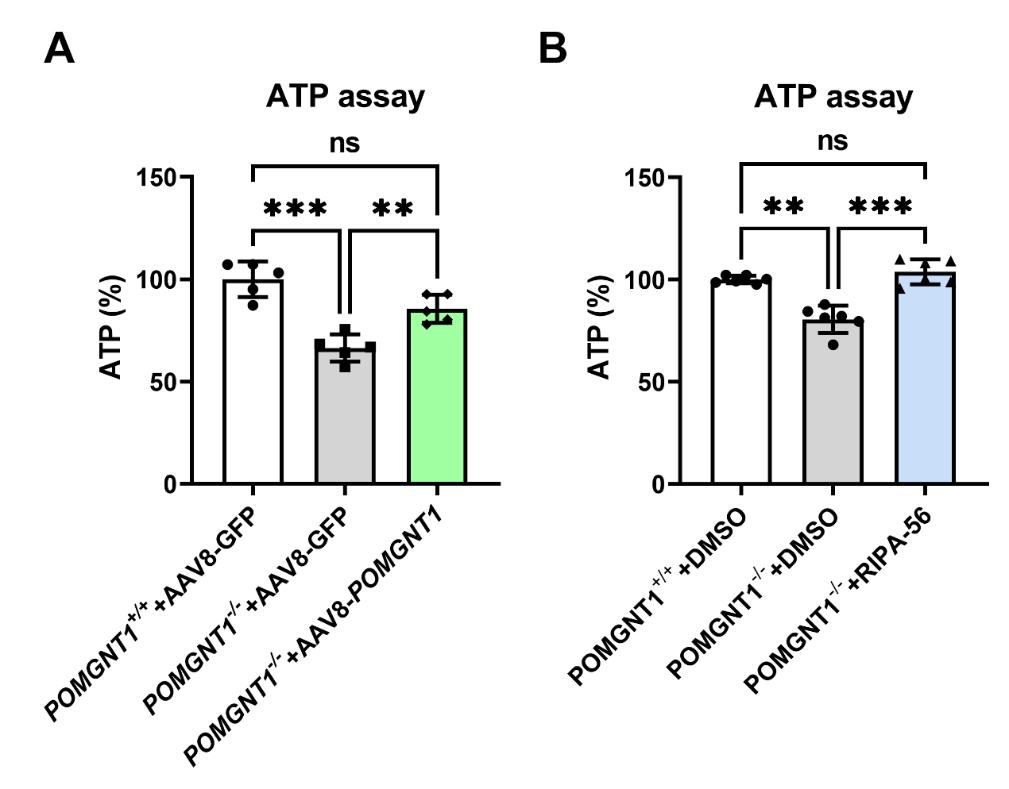


(Supplementary figure 5)

1. Quantitative analysis of ATP production in hRPE *POMGnT1* knockout (KO) cells after gene augmentation. The figure compares ATP levels in WT cells (normalized to 100%), KO cells treated with AAV8-GFP (negative control), and KO cells treated with AAV8-*POMGnT1*. The mean ATP levels were 100%±1.867, 66.43%±6.627, and 85.60%±6.914, respectively. The AAV8-*POMGnT1* group significantly restored ATP production compared to the AAV8-GFP KO control. (n=5; *, p<0.05; **, p<0.01; ***, p<0.001)
2. Quantitative analysis of ATP production in hRPE *POMGnT1* KO cells after RIPK1 inhibition. The figure compares WT cells (normalized to 100%), KO cells treated with DMSO (vehicle control), and KO cells treated with RIPA-56 (40 µM). The mean ATP levels were 100%±8.675, 80.56%±6.730, and 103.7%±6.092, respectively. The RIPA-56 group significantly restored ATP production compared to the DMSO vehicle control. (n=6; *, p<0.05; **, p<0.01; ***, p<0.001)

6. Evidence of necroptosis in the RPE layer in vivo demonstrated by RIP3 immunopositivity in *Pomgnt1^L120R/ L120R^* mice.


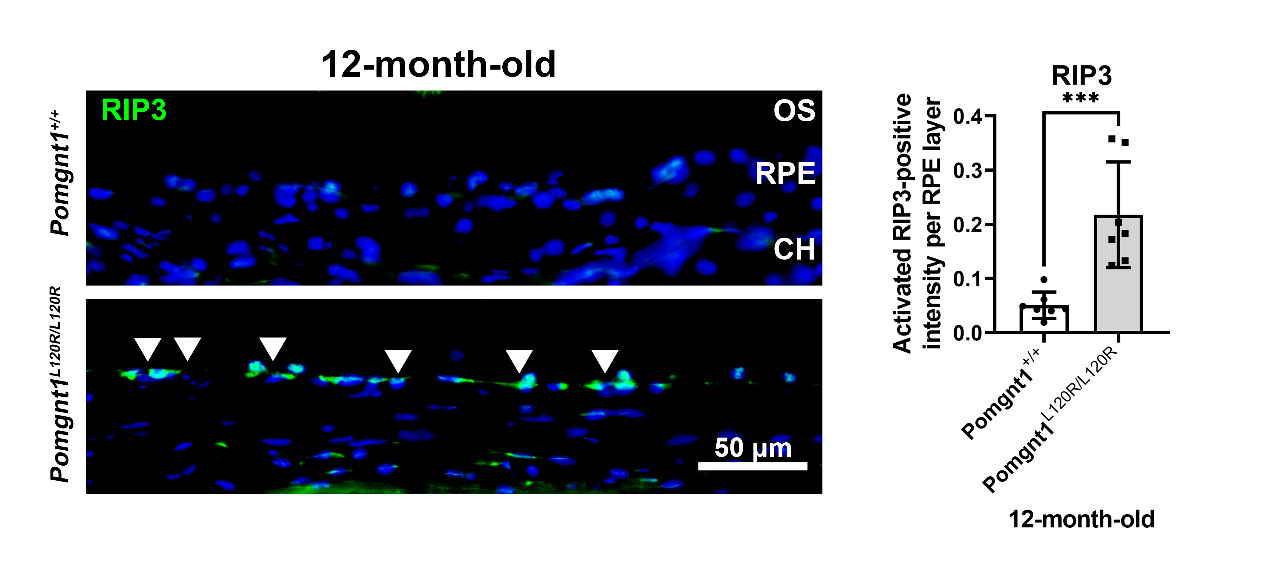


(Supplementary figure 6)

RIP3 immunopositivity in the RPE layer of *Pomgnt1^L120R/ L120R^* mice.

(A) Representative immunofluorescence images showing RIP3 positivity (green, arrow) specifically within the RPE layer of 12-month-old *Pomgnt1^+/+^* and *Pomgnt1^L120R/ L120R^* mice. Nuclei were counterstained with DAPI (blue). (OS: outer segment; RPE: retinal pigment epithelium, CH: choroid)

(B) Quantitative analysis of RIP3 immunopositivity intensity per RPE layer. The bar graph shows a significant increase in RIP3 protein levels in the RPE layer of *Pomgnt1^L120R/ L120R^* mice compared to controls. (n=7; *, p<0.05; **, p<0.01; ***, p<0.001)

7. Recombinant Protein Pull-Down Confirms Selective Disruption of the POMGnT1–ENO1 Interface by L120R mutation

To directly validate the protein–protein interactions suggested by our original Co-IP data, we generated recombinant human proteins carrying affinity tags using pBAD-His, pET28a-FLAG, and pGEX-6P-GST expression plasmids. The constructs included His-tagged POMGNT1 and the L120R mutant, FLAG-tagged S-arrestin, and GST-conjugated ENO1. Recombinant protein expression was induced with IPTG or L-arabinose, depending on each vector system, and pull-down assays were performed using a GST-based capture approach (#21516, Thermo, USA). After pull-down, Western blotting with anti-His (Cell Signaling #2365) and anti-FLAG (Abcam ab205606) antibodies revealed that GST-ENO1 robustly precipitated both S-arrestin and wild-type POMGNT1, demonstrating a direct biochemical interaction in vitro. In contrast, the POMGNT1-L120R variant was absent from the pull-down eluate, indicating that the L120R substitution disrupts binding between POMGNT1 and ENO1/S-arrestin.

To corroborate these findings in vivo, we performed retinal pull-down and co-immunoprecipitation assays using lysates from *Pomgnt1*^+/+^ and *Pomgnt1^L120R/L120R^* mice. Immunoprecipitation targeting endogenous POMGnT1 followed by SDS-PAGE and mass spectrometry identified ENO1 and S-arrestin (SAG) as physiological interacting partners (Figure 4). Complex formation among POMGnT1, ENO1, and SAG was detectable in both genotypes; however, when ENO1 was immunoprecipitated, POMGnT1 co-precipitated only in wild-type but not in *Pomgnt1^L120R/L120R^* retinal lysates, whereas SAG remained associated in both cases. Together, these in vitro and in vivo results provide convergent biochemical evidence that the L120R mutation selectively disrupts the POMGnT1–ENO1 interface.
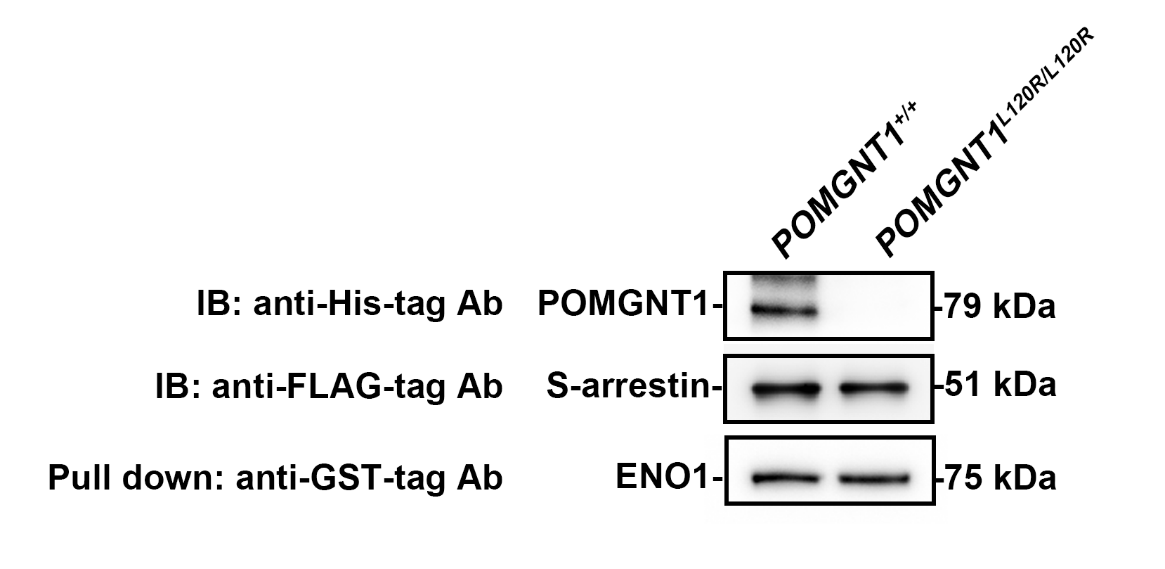


(Supplementary figure 7) **Direct GST pull-down assay confirms selective loss of POMGnT1–ENO1 binding caused by the L120R mutation.** Purified recombinant GST-ENO1, His-tagged POMGNT1 (WT), His-POMGNT1-L120R, and FLAG-S-arrestin were expressed in bacterial systems and subjected to a GST pull-down assay. Western blot analysis using anti-His and anti-FLAG antibodies revealed that GST-ENO1 precipitated S-arrestin and wild-type POMGNT1, confirming direct protein–protein interactions. In contrast, POMGNT1-L120R was absent from the pull-down eluate, indicating disruption of the ENO1–POMGNT1 interface. Retinal pull-down and co-immunoprecipitation assays from *Pomgnt1⁺/⁺* and *Pomgnt1ᴸ¹²⁰ᴿ/ᴸ¹²⁰ᴿ* mice further corroborated these findings, demonstrating that endogenous ENO1 co-precipitates with POMGnT1 only in wild-type retina, while S-arrestin remains associated in both genotypes.
