# Supplementary material for: Glycosylation-driven necroptosis in retinal degeneration: dual rescue by AAV8 gene therapy and RIPK1 inhibition
Source: Cell Death Discov. 2026 Apr 9;12:241. doi: 10.1038/s41420-026-03098-8 (PMC13187256; doi:10.1038/s41420-026-03098-8)
Supplement: Supplementary file 2 — Raw data gel blot [file 41420_2026_3098_MOESM2_ESM.pdf]

All unedited blot for Figure 3C

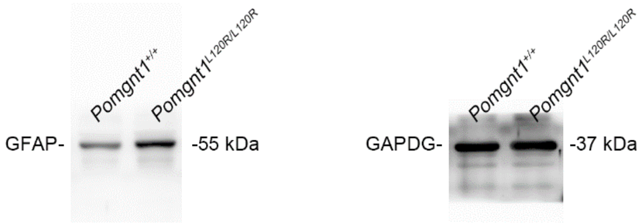

All unedited blot for Figure 5B

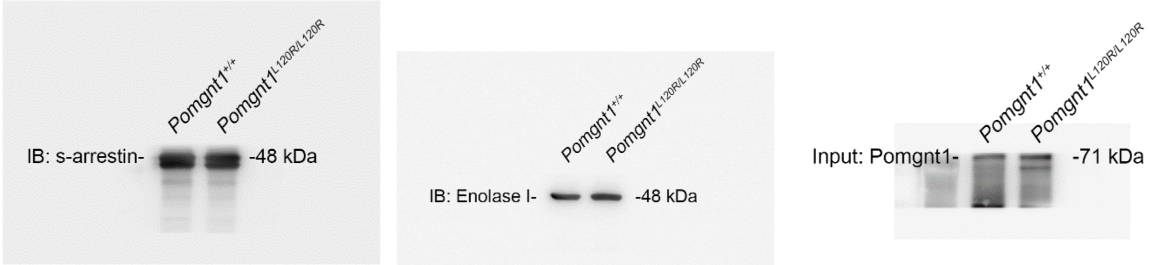

All unedited blot for Figure 5C

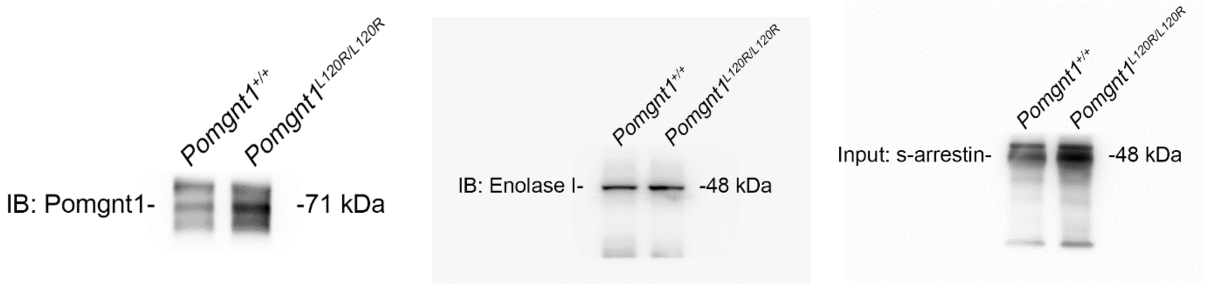

All unedited blot for Figure 5D

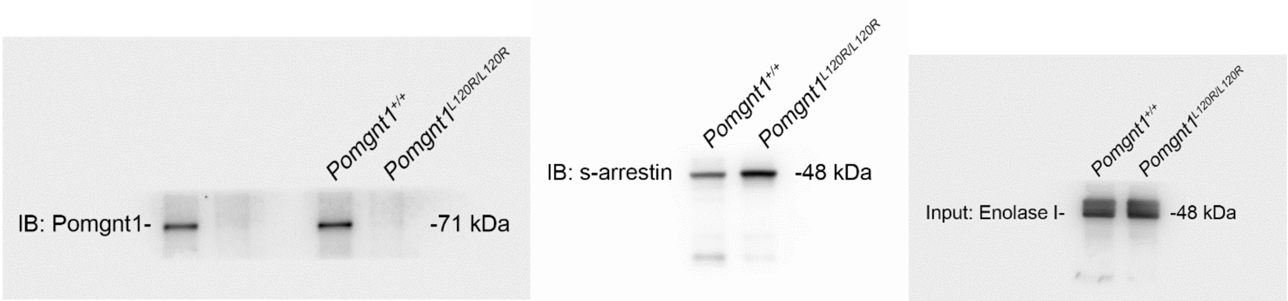

All unedited blot for Figure 6

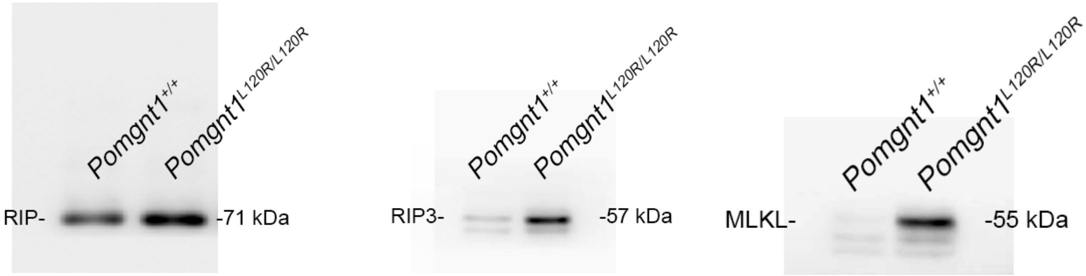

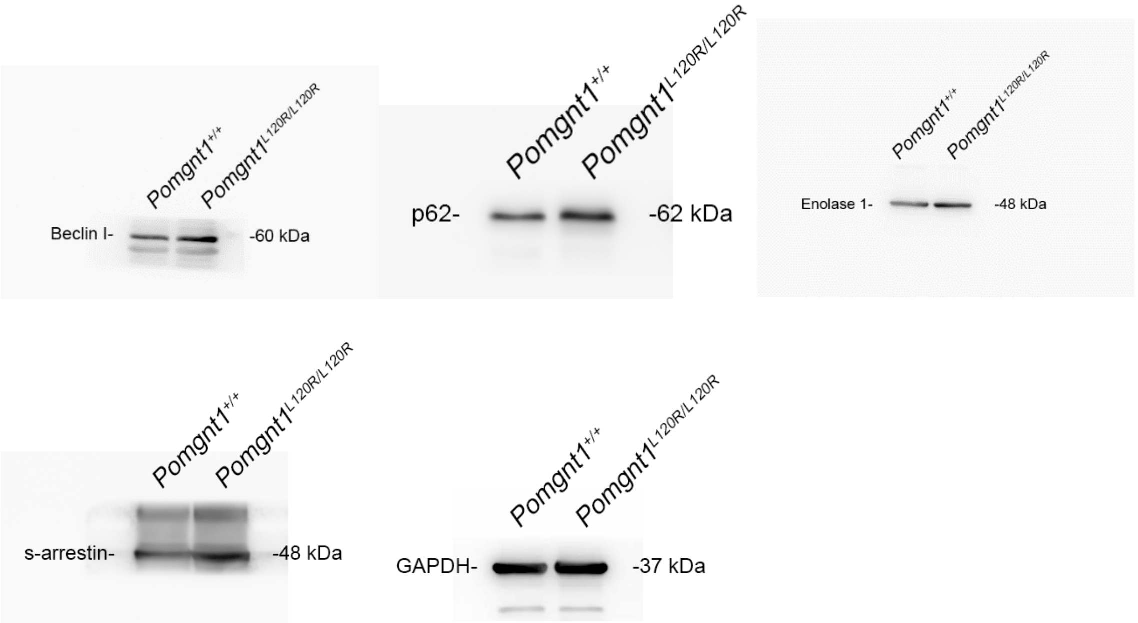

All unedited blot for Figure 7 (hRPE)

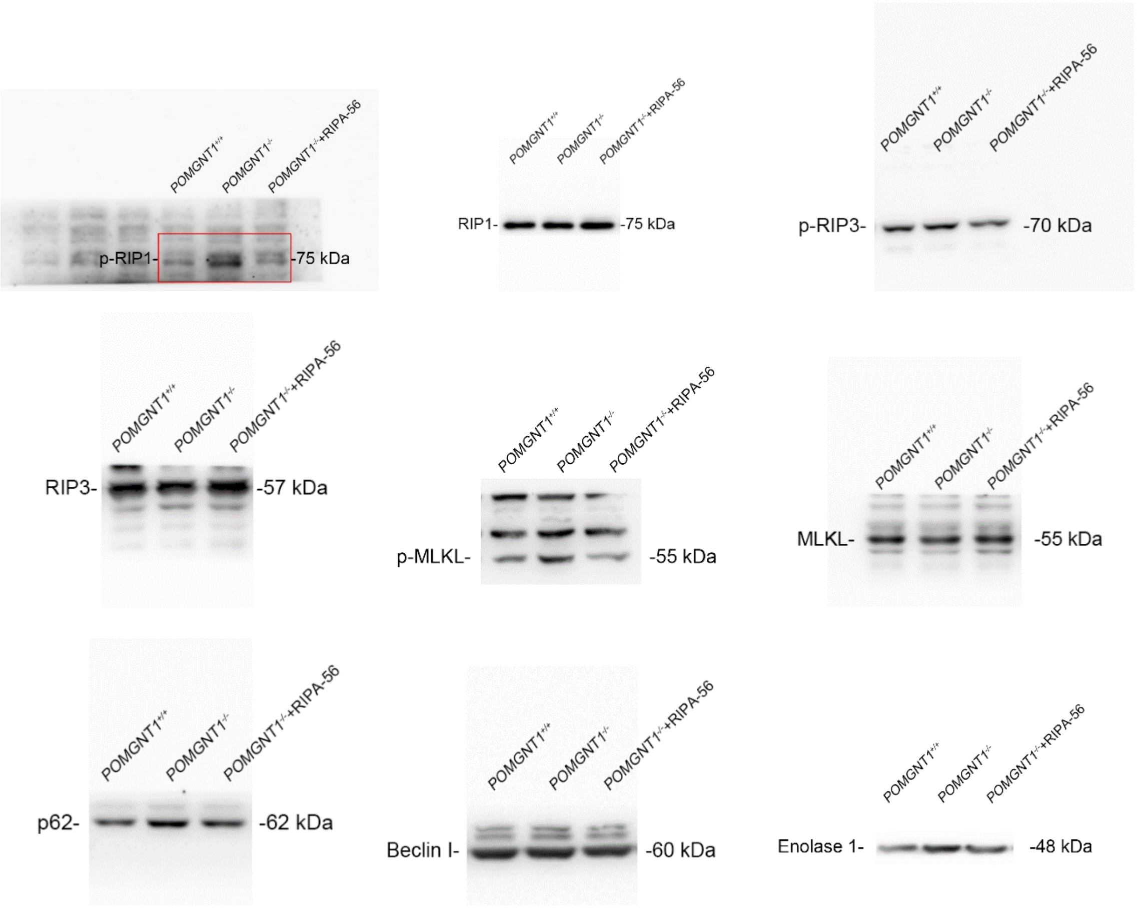

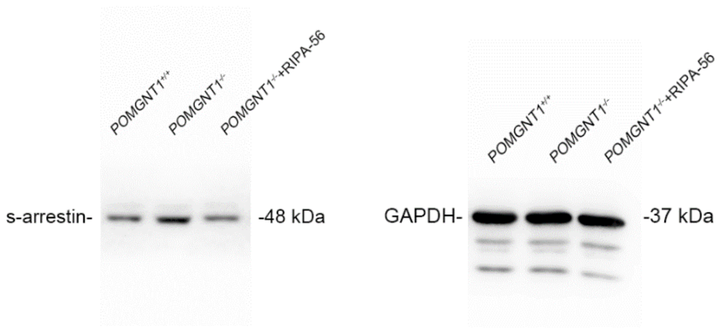

All unedited blot for Figure 8C

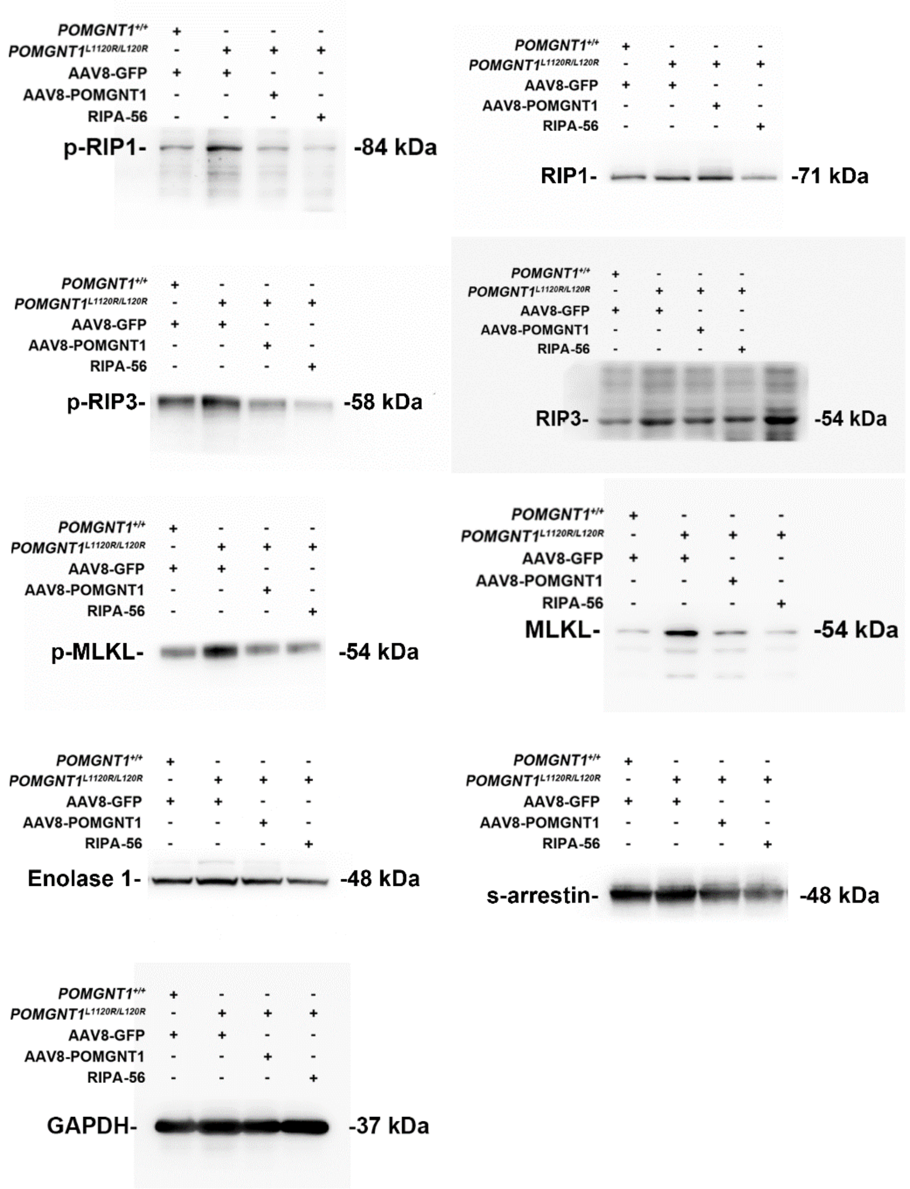

All unedited blot for Figure 8E

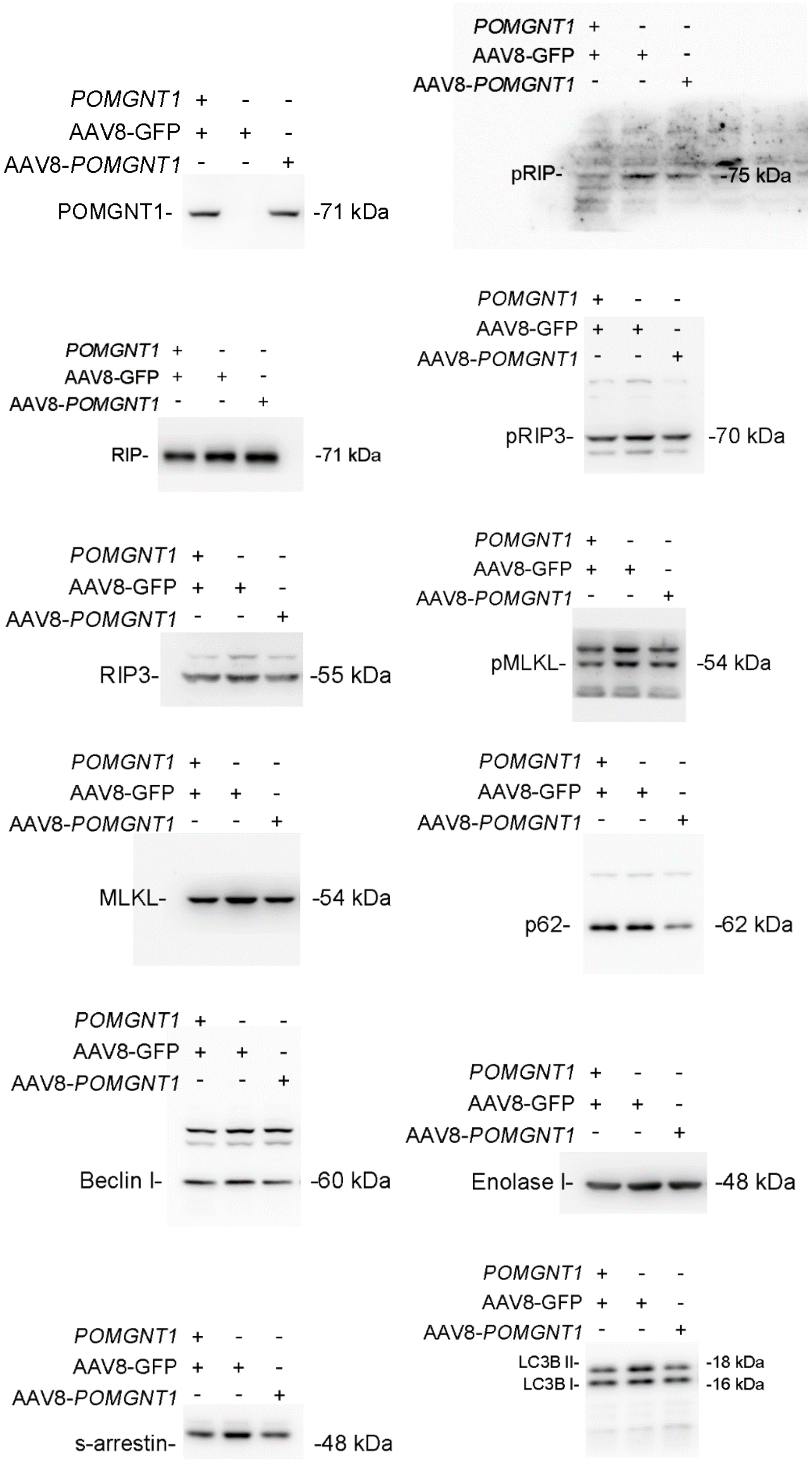

POMGNT1 + - -
AAV8-GFP + + -
AAV8-POMGNT1 - - +
POMGNT1-
-71 kDa
pRIP-
-75 kDa
RIP-
-71 kDa
pRIP3-
-70 kDa
RIP3-
-55 kDa
pMLKL-
-54 kDa
MLKL-
-54 kDa
p62-
-62 kDa
Beclin I-
-60 kDa
Enolase I-
-48 kDa
s-arrestin-
-48 kDa
LC3B II-
LC3B I-
-18 kDa
-16 kDa

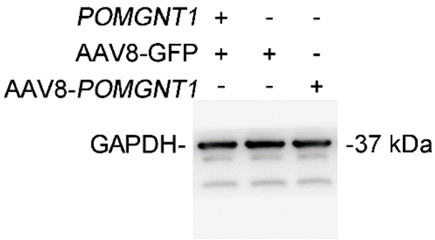

All unedited blot for Supplementary figure 1

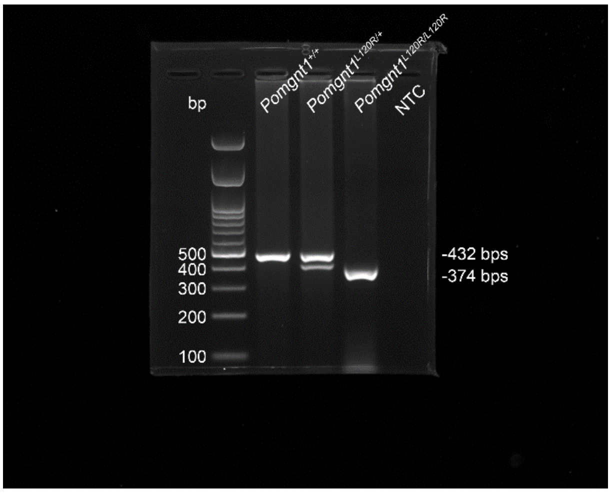

All unedited blot for Supplementary figure 7

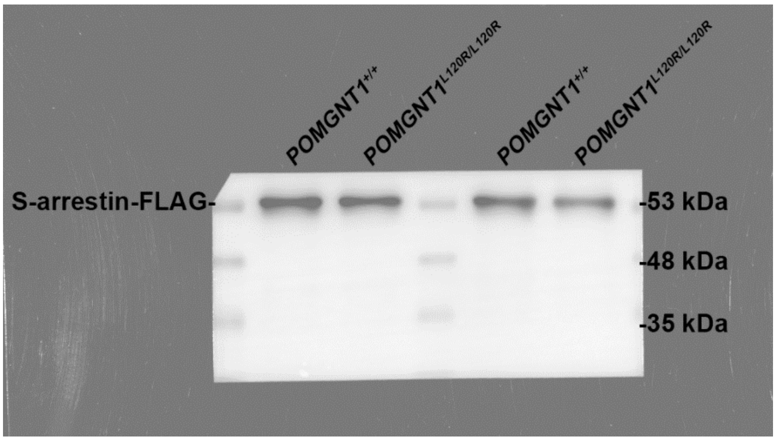

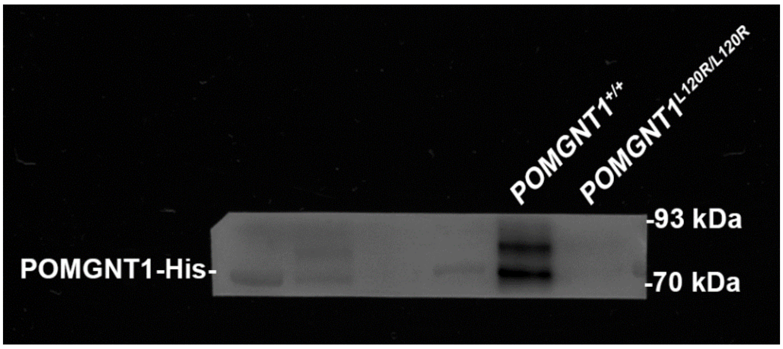

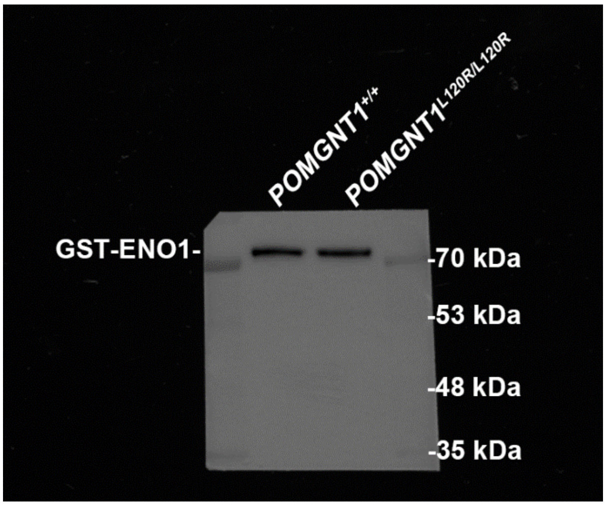

POMGNT1+/+
POMGNT1L120R/L120R
GST-ENO1-
-70 kDa
-53 kDa
-48 kDa
-35 kDa
